# A biometric survey of known and prospective murine models of posterior microphthalmia-nanophthalmia

**DOI:** 10.1016/j.exer.2025.110335

**Published:** 2025-03-26

**Authors:** Navdeep Gogna, Jai Pinkney, Lisa Stone, MHD Mustafa Khorzom, Fuxin Zhao, Gayle B. Collin, Juergen K. Naggert, Mark P. Krebs, Patsy M. Nishina

**Affiliations:** The Jackson Laboratory, 600 Main Street, Bar Harbor, ME, 04609, USA

**Keywords:** Posterior microphthalmia (microphthalmos), Nanophthalmia (nanophthalmos), MAC, Axial length, Ocular biometry, Corneal radius of curvature, Optical coherence tomography

## Abstract

Posterior microphthalmia and nanophthalmia are related genetic conditions that disrupt ocular growth. Here, we conducted a biometric analysis of mouse models to assess shared features of these diseases. Three known microphthalmia alleles (*Mfrp*^*rd6*^, *Prss56*^*glcr4*^, and *Adipor1*^*tm1Dgen*^) and two prospective alleles (*C1qtnf5*^*tm1.1(KOMP) Vlcg*^ and *Prss56*^*em2(IMPC)J*^) were introgressed onto the C57BL/6J (B6) genetic background and compared to B6 mice at 1 through 12 months of age. Biometric parameters obtained using optical coherence tomography were analyzed statistically to identify strain differences. Fundus imaging and histological analyses were performed to assess ocular morphology. *Mfrp*^*rd6*^, *Prss56*^*glcr4*^, and *Prss56*^*em2(IMPC)J*^ mice had significantly shorter axial and posterior lengths, and longer anterior chamber depth compared to controls at all ages studied. *Adipor1*^*tm1Dgen*^ mice exhibited similar, but less severe, biometric changes. Axial length was not significantly changed in *C1qtnf5*^*tm1.1(KOMP)Vlcg*^ mice, but reduced anterior chamber depth and increased lens thickness were observed at one month of age. Lens and corneal thicknesses were otherwise unchanged in the models as compared to B6 controls. Corneal radius of curvature, examined at 4 months of age, was significantly decreased in all models relative to controls. Micropthalmia was observed independent of retinal degeneration (*Mfrp*^*rd6*^, *Adipor1*^*tm1Dgen*^) or retinal thickening (*Prss56* mutants). *Prss56* mutants developed retinal folds that were absent from other mutants and controls. We conclude that, in mice, *Mfrp*, *Prss56*, and *Adipor1* mutations yield similar microphthalmia phenotypes involving both the anterior and posterior eye. Changes to anterior chamber depth, lens thickness, and corneal curvature in *C1qtnf5*^*tm1.1(KOMP)Vlcg*^ mice suggest a role of *C1qtnf5* in anterior ocular growth.

## 1. Introduction

Microphthalmia is a heterogeneous developmental disease characterized by a small eye size at birth, affecting approximately 1 in 7000 individuals (Jackson and Moosajee, 2023). Microphthalmia is often accompanied by coloboma, anterior segment dysgenesis, cataract, or vitreoretinal dysplasia, resulting in a disease spectrum that includes microphthalmia, anopthalmia (no eyes), and coloboma (MAC) (Jackson and Moosajee, 2023; Skalicky et al., 2013). More rarely, shortening of the posterior segment (posterior micropthalmia) or both the anterior and posterior segments (nanophthalmos) are observed without severe ocular malformations. The allelic overlap of these conditions (Aldahmesh et al., 2011) has led to the classification of posterior microphthalmia-nanophthalmia as a microphthalmia subtype (Nowilaty et al., 2013) herein designated PMN. In MAC, vision can be severely disrupted depending on the extent of ocular tissue dysgenesis (Skalicky et al., 2013), while in PMN vision is generally better, but may become compromised due to hyperopic refractive error (farsightedness), angle-closure glaucoma and serous retinal detachment (Niazi et al., 2024; Sundin et al., 2005). Nearly all cases of microphthalmia (98 %) are linked to genetic variation; so far, variants in 98 genes underlie both syndromic and non-syndromic forms of these diseases (Harding and Moosajee, 2019; Jackson and Moosajee, 2023). Current efforts to understand microphthalmia pathogenesis are focused on the cellular and molecular processes by which these disease-associated genes influence ocular growth.

Critical insights into ocular growth may be obtained from studies of PMN genes. Of the 98 genes associated with small eyes, only six (*BEST1, CRB1, MFRP, MYRF, PRSS56,* and *TMEM98*) are associated with PMN (Carricondo et al., 2018; Jackson and Moosajee, 2023), leading to the hypothesis that shared molecular processes are affected by variants in these genes. Further, *MFRP, PRSS56,* and *TMEM98* variants are associated with both microphthalmia, resulting in a decreased axial length, and myopia, resulting in an increased axial length (Jackson and Moosajee, 2023), which may indicate common molecular functions. Moreover, within the eye, *BEST1*, *MFRP*, *MYRF,* and *TMEM98* show expression prominently in the retinal pigment epithelium (RPE) and ciliary body (Cross et al., 2020; Esumi et al., 2009; Kameya et al., 2002; Marmorstein et al., 2000). Thus, the six PMN genes may function in shared molecular processes targeting these tissues.

Studies of mouse PMN models (Harding et al., 2021) are likely to provide important clues to disease pathogenesis. Several models have phenotypes similar to those observed in patients bearing variants in the orthologous genes. In a study that identified *PRSS56* as a microphthalmia gene, a statistically significant 2.7–5.0 % decrease in axial length and equatorial diameter was observed in eyes of *Prss56*^*grm4*^ (also known as *Prss56*^*glcr4*^) mice relative to controls at postnatal day 13 (P13) and older (Nair et al., 2011). A *Prss56* knockout mouse, *Prss56*^*Cre*^ (Jourdon et al., 2016) showed similar effects (Paylakhi et al., 2018). *Mfrp*^*rd6*^ mice were presented as a model of retinal degeneration (Hawes et al., 2000), and were found initially to lack a robust PMN phenotype (Cross et al., 2019; Fogerty and Besharse, 2011; Sundin, 2005; Sundin et al., 2005). Later studies detected a small but statistically significant decrease in axial length due to the *Mfrp*^*rd6*^ mutant allele (Gogna et al., 2022; Koli et al., 2021), and a qualitative decrease in ocular size was reported among mice carrying an *Mfrp* c.498_499insC knock-in allele (Chekuri et al., 2019). In contrast, ablation of *Myrf* in the RPE of conditional *Myrf* knockout mice did not yield significant changes in axial length or other axial parameters, although evidence of RPE pathology and retinal degeneration was present (Garnai et al., 2019). Likewise, no significant change in axial length was observed when missense mutations associated with human *TMEM98* nanophthalmia were introduced into the mouse *Tmem98* gene; however, outer retinal folds were observed (Cross et al., 2019; Hassall et al., 2023). A decrease in ocular size has not yet been reported in *Crb1* or *Best1* mutant mice.

A recurring issue in these studies is that the effects of homozygous PMN gene mutations on axial length in mice are relatively much smaller than in the human nanophthalmia caused by variants of orthologous genes, making a reduced axial length difficult to detect (Cross et al., 2019; Garnai et al., 2019; Gogna et al., 2022; Hassall et al., 2023; Koli et al., 2021; Nair et al., 2011; Paylakhi et al., 2018; Sundin, 2005; Sundin et al., 2005). Thus, while studies of PMN genes have recapitulated human *MFRP* and *PRSS56* phenotypes, identifying and characterizing the molecular roles of additional PMN genes in mice may require precise methods to measure ocular size and the use of large cohorts (Koli et al., 2021; Nair et al., 2011; Pardue et al., 2013; Paylakhi et al., 2018).

Here, to address these concerns, we performed ocular biometry, including axial measures of tissue thicknesses, and chamber depths, on mouse PMN models at 1 through 12 months of age. Importantly, we included the corneal radius of curvature as a measure of anterior ocular size. Our major objective was to determine whether ocular biometry phenotypes were similar amongst the models. We examined ocular growth in models on a common B6 genetic background bearing known alleles of *Mfrp*, *Prss56,* and *Adipor1* and prospective PMN alleles of *C1qtnf5* and *Prss56.* Our results reveal shared PMN features in the *Mfrp*, *Prss56,* and *Adipor1* models and suggest *C1qtnf5* influences anterior ocular growth.

## 2. Materials and methods

### 2.1. Animals

Mouse strains and the abbreviations used in this study for them are listed in Supplementary Table ST1. Mice were homozygous for their respective mutant alleles, and were bred and maintained under standardized conditions of 12:12 light:dark in the Research Animal Facility at The Jackson Laboratory (JAX). Mice maintained in pressurized individual ventilation caging were provided with LabDiet 5K52 and HCl-acidified water (pH 2.8–3.2) *ad libitum* and monitored for the maintenance of a pathogen-free environment. All strains were generated on a C57BL/6J (B6) genetic background or backcrossed to B6 for at least five generations prior to characterization, and confirmed to be free of the *Crb1*^*rd8*^ mutation. Animal protocols were approved by the JAX Institutional Animal Care and Use Committee (AUS99089) and complied with guidelines set forth by the ARVO Animal Statement for the Use of Animals in Ophthalmic and Vision Research.

### 2.2. Genotyping

Genomic DNA was extracted from either tail tips (*<*2 mm) or ear punches using a standard protocol (Gogna et al., 2022). Briefly, tissue was incubated in 50 mM sodium hydroxide solution for 30–50 min at 95 ^◦^C, followed by the addition of 1M Tris HCl neutralizing reagent buffer (final concentration 200 mM), vortexed and spun at 3600 rpm for 8 min at 4^◦^C. For PCR amplification, 1 μl supernatant was used in an allele-specific assay to determine genotype of *Prss56*^*glcr4*^ mice*. Mfrp*^*rd6*^*, Adipor1*^*tm1Dgen*^, *Prss56*^*em2(IMPC)J*^ and *C1qtnf5*^*tm1.1(KOMP)Vlcg*^ mice were genotyped by the JAX Transgenic Genotyping Service. PCR primer sequences are listed in Supplementary Table ST2.

### 2.3. RNA isolation and quantitative real-time (qRT) PCR

RNA was isolated from whole eyes or posterior eye cups as described previously (Collin et al., 2015) using an RNeasy Micro Kit (QIAGEN, MD, USA). Briefly, cDNA was synthesized by reverse transcription of RNA using Superscript IV First Strand cDNA Synthesis kit (Thermo Fisher Scientific, Inc., MA, USA) according to the manufacturer’s protocol. qRT-PCR was performed using an established manufacturer’s protocol with iTaq Universal SYBR Green SuperMix (Bio-Rad, CA, USA), gene-specific primers (Supplementary Table ST2) and a CFX96 Real-Time PCR Detection system (Bio-Rad). The comparative CT method (ΔΔCT) was applied to calculate a relative-fold change in transcripts and quantified using 2^−ΔΔCT^ with β-actin as an internal calibrator.

### 2.4. Axial and retinal dimensions

Ocular biometry was determined by optical coherence tomography (OCT) using a Bioptigen Ultrahigh-resolution (UHR) Envisu R2210 SD-OCT imaging system (Leica Microsystems, IL, USA). We modified a previously described approach to measuring axial parameters in mice by OCT (Pardue et al., 2013; Park et al., 2012) to provide sharp signals on our instrument (see Section 3.2). Briefly, mice were weighed and anesthetized with a ketamine/xylazine mixture (1.6 mL ketamine (100 mg/mL), 1.6 mL xylazine (20 mg/mL), and 6.8 mL sodium chloride (0.9 % w/v), injected intraperitoneally at 0.10 mL/20 g of body weight. A drop of GenTeal Severe gel (Alcon, TX, USA) was applied to both the eyes of mice to prevent corneal drying prior to image acquisition. Cross-sectional images were obtained using 1000 A-scans (single depth profile composed of time-gated reflections) per B-scans (frame composed of array of A-scans); 20 B-scans were acquired (1000 × 1 × 20). Images were converted to .tif format using the OCT Reader plugin for ImageJ provided by the OCT instrument manufacturer, registered, averaged and analyzed in Fiji (Schindelin et al., 2012). Pixel dimensions measured along or parallel to the OCT imaging axis were converted to physical units using the manufacturer’s default mean group refractive index for ocular tissue in mice (1.38). The resulting values were divided by refractive index values for each ocular segment [cornea, 1.4015; anterior chamber, 1.3336; lens, 1.45; vitreous, 1.3329; retina, 1.38] (Painter et al., 2024) to yield corrected physical dimensions for further analysis. SD-OCT B-scans were randomized, with age and strain identifiers removed prior to analysis of ocular dimensions.

To characterize retinal folds, volume scans (1000 × 100 × 10, 1.9 mm diameter) were obtained, and images were processed in Fiji using the methodology described previously (Krebs, 2017; Krebs et al., 2016).

### 2.5. Corneal radius of curvature measurement

For measurement of corneal radius of curvature (CRC), a telecentric lens was used, which is uniquely suited for measuring corneal surfaces (Liu et al., 2020). OCT images were calibrated to obtain the pixel-to-mm conversions in x, y and z dimensions, similar to the approach described previously (Liu et al., 2020). For x and y dimensions, a dual axis linear scale micrometer (Edmund Optics, #58608) was imaged. Pixel-to-mm conversion was obtained by dividing the overall length of the imaged micrometer by the number of pixels in the OCT image along that axis. That is, by dividing the horizontal (x) and vertical (y) distance across a 2-mm square defined by the micrometer by the number of pixels in the OCT image along those axes (573 and 562 pixels along the x and y dimensions respectively). For the z dimension, we imaged a sharply reflecting object (corneal surface) at two positions along the optical axis (z axis) of the imaging system. The physical distance between the two positions (1.5 mm) was then divided by the number of pixels (714) separating the object reflection in the two images. For our system, x, y, and z calibrations were 3.49, 3.56, and 2.10 μm/pixel, respectively. To obtain CRC values, we used the trigonometric derivation CRC = (AD/2) + (ACW^2^/(8 X AD)) described previously (Tkatchenko et al., 2010); except that AD was measured as the distance between the anterior surface of the cornea and the anterior pole of the lens, and ACW was measured as a horizontal line at the anterior pole of the lens between the outer surfaces of the cornea. We also verified our CRC values by using Fiji to fit a circle to intensity maxima along the corneal surface as shown in Supplementary Fig. S1. This approach indicates that the corneal surface in cross-section was well approximated by a circle, validating the use of the Tkatchenko et al. equation. Values obtained from either method differed by less than 1 %. The accuracy of both methods was tested by measuring the radius of curvature of calibration spheres (purchased from McMaster-Carr, New Jersey). The spheres measured included a 3/32-inch diameter (2.381 mm) and a 1/8-inch diameter (3.175 mm) silicon-nitride ceramic balls and a 3 mm diameter tungsten carbide ball (all spheres had a diameter tolerance *<*0.0013 mm). OCT images of these spheres were acquired and processed using parameters identical to those used for imaging mice, yielding a radius of curvature for each calibration sphere that was within 1 % of the manufacturer’s specified dimensions.

### 2.6. Statistical analysis

Data analysis was performed using JMP (SAS Institute, Cary, NC, USA) and GraphPad Prism (GraphPad Software, San Deigo, CA, USA) statistical analysis software. Mixed model analysis of variance (ANOVA), followed by Dunnett’s multiple comparison post-hoc test was used to identify significant differences in AL and other ocular parameters between different mouse strains at 1, 4, 8, and 12 months of age using GraphPad Prism. Results were validated by factorial ANOVA and three-way factorial analysis of covariance (ANCOVA) using the fit model function in JMP. AL was selected as a dependent variable, and main and interactive effects of three independent, categorical variables of mouse strain, age and gender were examined. Body weight was used as a covariate. ANCOVA assumptions were tested; linear relationship between body weight and AL as well as no interaction between the covariate and independent variables were confirmed. Data were tested for normal distribution and equal variance. The F ratio (ratio of the variation explained by the model and the unexplained variation; [Prob *>* F] *<* 0.01), adjusted R^2^ (goodness-of-fit measure between the model and the dependent variable), and root mean square error (RMSE) were calculated and compared with and without the inclusion of covariate. The RMSE measures the error of a model in predicting quantitative data and indicates how accurately the model predicts the response. Pearson’s correlation and linear regression analyses were also performed.

### 2.7. Fundus imaging

Fundus images were acquired using a Micron IV fundus camera (Phoenix Research Laboratories, Pleasanton, CA, USA) and digitally processed as previously described (Krebs et al., 2016), except that 1 % cyclopentolate (Akorn) or 1 % atropine (Akorn) was used as the dilating agent and mice were anesthetized with isoflurane provided by a vaporizer (Kent Scientific, Torrington, CT, USA).

### 2.8. Refractive error measurement

The refractive state was measured with a custom-built automated eccentric infrared photo refractor and calibrated according to a previously published protocol (Schaeffel et al., 2004). Each eye was assessed three times. No statistically significant difference was observed between the right and left eye (statistical significance determined by paired two-tailed Student’s t-test), thus, values from both eyes of each animal were averaged.

### 2.9. Immunohistochemistry (IHC) and histology

The protocol for immunohistochemical assays was previously described (Naggert et al., 2022). Briefly, eyes were enucleated after carbon dioxide asphyxiation, oriented in a cryomold filled with optimal cutting temperature compound, snap-frozen in a metal box containing 2-methyl butane by immersion in liquid nitrogen, and cryosectioned (10-μm thickness). For staining, sections were fixed with 4 % paraformaldehyde in phosphate-buffered saline (PBS) for 10 min, incubated in blocking solution at room temperature for 30 min and incubated with anti-glial fibrillary acidic protein (GFAP) (Dako Z0334; 1:200) overnight. After washing in PBS, tissues were incubated in secondary antibody (Alexa Fluor 555–conjugated donkey anti-rabbit (Invitrogen, A31572); 1:200) and 4′,6-diamidino-2-phenylindole (DAPI; Invitrogen, D1306; 1:600) for 2 h at room temperature. After washing in PBS, samples were mounted in Vectashield (Vector Laboratories, EW-93952–23) and fluorescent signals visualized using an Axio Observer.z1 fluorescence microscope with an ApoTome 2.0 accessory (Carl Zeiss, Germany). For negative controls, the primary antibody was omitted. For histology, enucleated eyes were fixed in acetic acid:methanol:PBS (1:3:4), followed by paraffin embedding and sectioning at 5 μm. Tissues were then de-paraffinized, stained with hematoxylin and eosin, and slides scanned using a NanoZoomer 2.0-HT digital slide scanner (Hamamatsu, Shizuoka, Japan). Histology images were captured at 40x magnification using NDP.view2 viewing software (Hamamatsu, Shizuoka, Japan).

## 3. Results

### 3.1. Strain selection and gene expression

To identify shared features of microphthalmia among genetic models, we selected mice bearing *Prss56*^*glcr4*^, *Mfrp*^*rd6*^, and *Adipor1*^*tm1Dgen*^ alleles (Gogna et al., 2022; Paylakhi et al., 2018; Rice et al., 2015). We also examined two prospective PMN models generated by the International Mouse Phenotyping Consortium (IMPC) (Lloyd, 2024) at The Jackson Laboratory (JAX). The first, homozygous for a *Prss56* knockout allele (*Prss56*^*em2(IMPC)J*^), bears a deletion of exons 3 and 4 in *Prss56,* produced using CRISPR- Cas9 mediated genome editing (Elrick et al., 2024). The second bears a *C1qtnf5* knockout allele (*C1qtnf5*^*tm1.1(KOMP) Vlcg*^) created by a *lacZ* reporter gene replacement of *C1qtnf5* exons 2 and 3. The *C1qtnf5* knockout model was included because this gene may function with *Mfrp* in the same molecular pathways, as it is dicistronic with *Mfrp* (Chavali et al., 2011; Kameya et al., 2002), and C1QTNF5 and MFRP have been shown to interact (Mandal et al., 2006; Shu et al., 2006). An effect of *C1qtnf5* on ocular growth has not yet been reported in *C1qtnf5*^*Ser163Arg*^ knock-in mice (Chavali et al., 2011; Shu et al., 2006) or an independent *C1qtnf5* knockout strain (Borooah et al., 2023). Importantly, all mutations in this study were previously generated using B6 mice or introgressed onto B6 by repeated backcrossing, thereby minimizing the potential for differences in genetic background among the microphthalmic models. All alleles were homozygous in the strains studied.

**Fig. 1.**
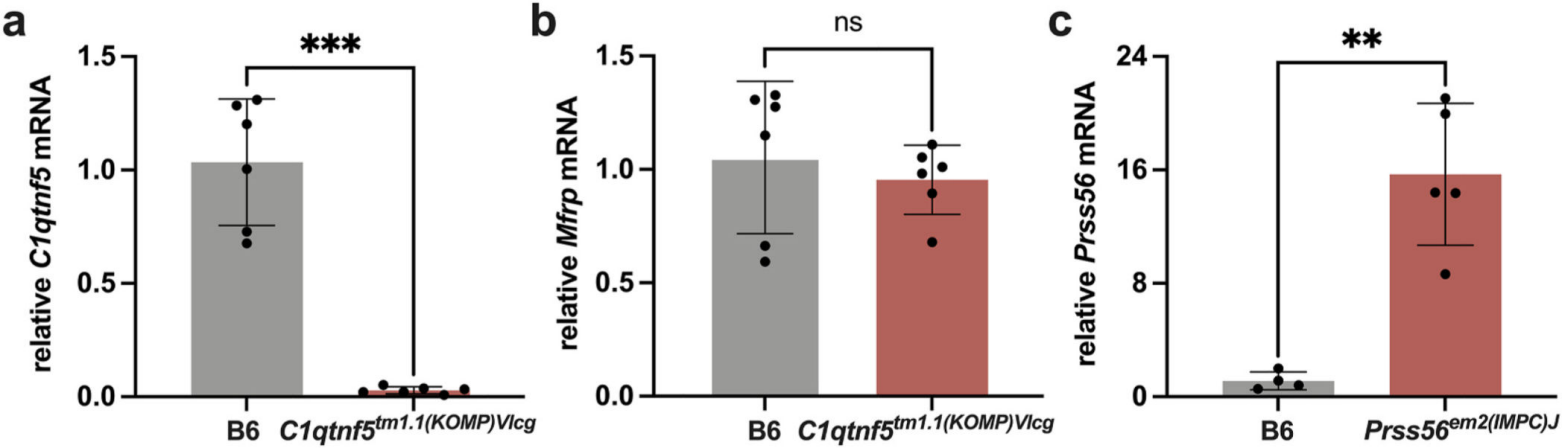
Expression analysis by qRT-PCR. **a-b.** qRT-PCR analysis of *C1qtnf5*^*tm1.1(KOMP)Vlcg*^ and B6 control posterior eyecups at 1.5 months of age. **a.** A statistically significant reduction in *C1qtnf5* expression was observed in posterior eyecups of *C1qtnf5*^*tm1.1(KOMP)Vlcg*^ mice compared to B6 controls. **b.** No significant change in *Mfrp* expression was seen in *C1qtnf5*^*tm1.1(KOMP)Vlcg*^ eyecups when compared with B6 controls. n = 6 for each strain, both sexes included. **c.** qRT-PCR of cDNA from whole eyes at two months of age showed a significant upregulation of *Prss56* expression in *Prss56*^*em2(IMPC)J*^ mice compared to B6 controls, n = 4–5 for each strain. Values indicate mean ± SD. **p < 0.005; ***p < 0.001; ns – non-significant.

The expression of target gene transcripts and encoded proteins in the *Prss56*^*glcr4*^, *Mfrp*^*rd6*^, and *Adipor1*^*tm1Dgen*^ models has been characterized previously (Kameya et al., 2002; Nair et al., 2011; Rice et al., 2015). To assess the effects of the targeted mutations on transcript levels in the prospective IMPC knockout models, we performed quantitative RT-PCR on ocular cDNA (Fig. 1). *C1qtnf5* mRNA expression was significantly reduced in *C1qtnf5*^*tm1.1(KOMP)Vlcg*^ mutants compared to B6 controls, possibly due to nonsense-mediated decay (Fig. 1a). Since *Mfrp* and *C1qtnf5* are dicistronic, we examined *Mfrp* mRNA expression in *C1qtnf5*^*tm1.1(KOMP)Vlcg*^ mutant eyecups and found no change at 1.5 months of age (Fig. 1b). Interestingly, *Prss5*6 mRNA expression was upregulated in the whole eyes obtained from *Prss56*^*em2(IMPC)J*^ mice at two months of age (Fig. 1c), as observed in other *Prss56* mutants (Koli et al., 2021; Nair et al., 2011; Paylakhi et al., 2018). Similar to *Mfrp*^*rd6*^ and *Adipor1*^*tm1Dgen*^ mutants (Gogna et al., 2022), both *Prss56*^*glcr4*^ and *Prss56*^*em2(IMPC)J*^ strains showed significant changes in refraction at 10 weeks of age (Supplementary Fig. S2), when the refractive error increased and shifted towards hyperopia. The alleles, respective mutations, and effects on transcript levels for all the strains studied in this paper are listed in Table 1.

### 3.2. Overview of axial and retinal measurements

**Fig. 2.**
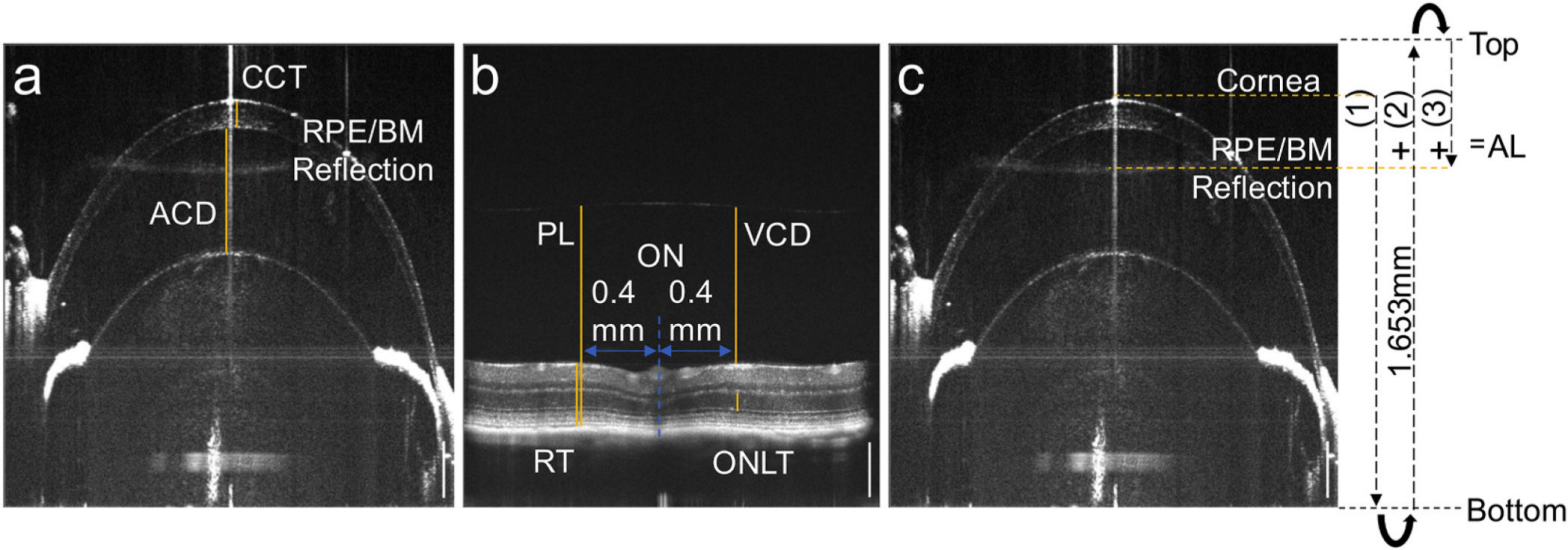
Representative full-height *in vivo* SD-OCT images from one eye of a B6 mouse. **a.** Ocular biometric parameters measured in the anterior eye include ACD and CCT. A reflection corresponding to the RPE and choroid, which includes the RPE/BM interface, was observed in the anterior image. **b.** Ocular biometric parameters measured in the posterior of the same eye include RT, ONLT, VCD, and PL. These parameters were each measured at a distance of 0.4 mm on either side of the optic nerve head (ONH) and the two values averaged. **c.** OCT image and corresponding schematic indicating the measurement of AL using a modified image folding approach (Park et al.). Curved arrows in the schematic indicate folding of the image. Due to double folding, the RPE/BM is observed in the same imaging window and with the same orientation as anterior tissues. AL is the sum of three measures: the distance from the front of the cornea to the bottom of the imaging window (1), the full height of the imaging window, determined to be 1.653 mm based on the default mean grouped refractive error (2), and the distance from the top of the image window to the RPE/BM reflection (3).

To determine the effect of the mutant alleles on ocular size, we used OCT ocular biometry to determine axial length (AL) and additional ocular parameters, including central anterior chamber depth (ACD), central corneal thickness (CCT), lens thickness (LT), vitreous chamber depth (VCD), outer nuclear layer thickness (ONLT), retinal thickness (RT), and posterior length (PL) (Fig. 2a and b). The imaging depth of our instrument (1.653 mm) was insufficient to capture the full AL of the eyes examined in a single image, so we initially tried to extend the effective imaging depth by simultaneously aquiring a primary image and a singly folded, inverted image as described (Pardue et al., 2013; Park et al., 2012). However, this approach resulted in blurred reflections on our instrument; therefore, we used a doubly folded image, which yielded sharper reflections (Fig. 2c). AL was calculated as the combined distance from the anterior corneal reflection, the full image depth, and the RPE/BM interface (Fig. 2c). The remaining parameters were obtained from two images as shown (Fig. 2a and b). To measure LT, the sum of ACD, CCT and PL was subtracted from AL. Previous mouse studies have variably defined VCD as the distance from the back of the lens to either the vitreal surface of retina (Chou et al., 2011) or to the RPE/BM (Tkatchenko et al., 2010). We defined VCD as the distance to the vitreal surface. As the position of the vitreal surface and therefore, the VCD are sensitive to changes in RT, we defined a second posterior measure, PL, as the distance from the back of the lens to RPE/BM. Since there is no clear RPE boundary at the optic nerve, the measurements for ONLT, RT, VCD, and PL were made at a distance of 0.4 mm on either side of the optic nerve head center and averaged (Fig. 2b).

### 3.3. Comparative analysis of AL

**Fig. 3.**
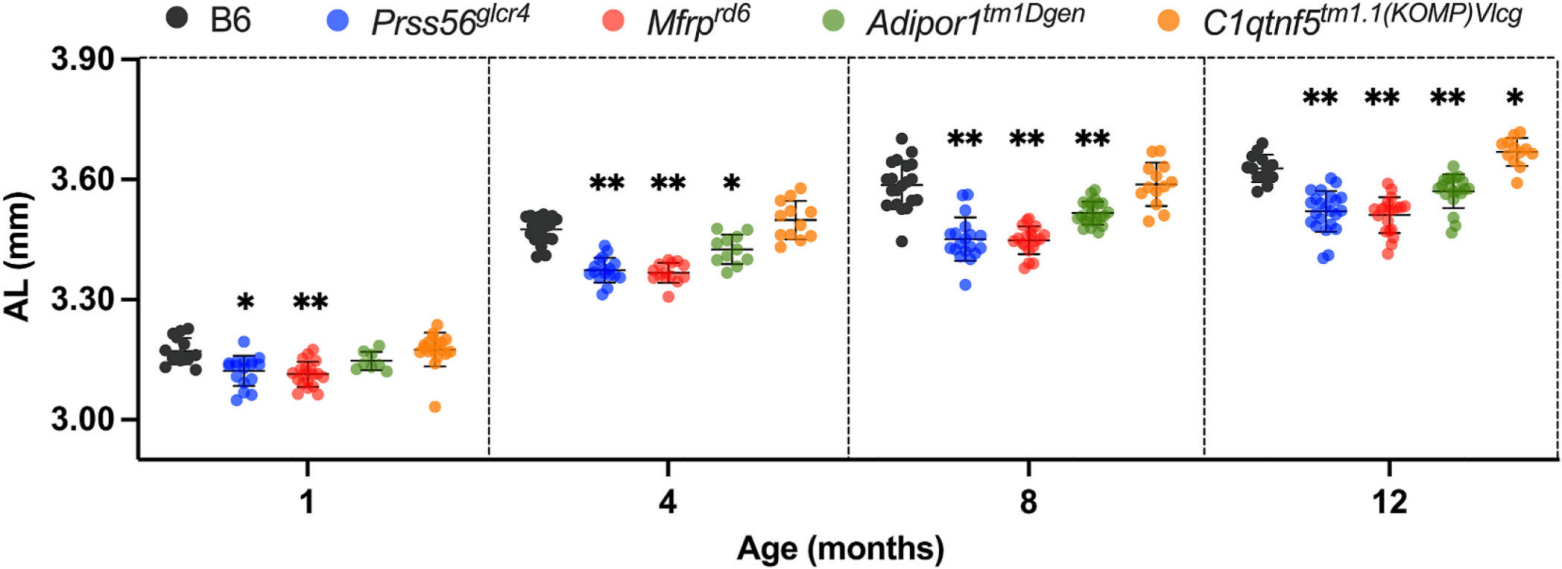
Comparative analysis of AL in B6 control and PMN models at 1, 4, 8, and 12 months of age. As compared to B6 controls, a progressive and significant decrease in AL was observed in *Prss56*^*glcr4*^, *Mfrp*^*rd6*^, and *Adipor1*^*tm1Dgen*^ mice. A small but significant decrease in AL was also observed in *C1qtnf5*^*tm1.1 (KOMP)Vlcg*^ mice at 1 month of age. B6, *Prss56*^*glcr4*^, *Mfrp*^*rd6*^, *Adipor1*^*tm1Dgen*^, and *C1qtnf5*^*tm1.1(KOMP)Vlcg*^ mice are represented with gray, blue, red, green, and orange circles, respectively. Mean ± SD values are indicated. The differences, whenever significant, as compared to B6, are indicated with asterisks above each mutant: *p < 0.05; **p < 0.001; n = 10–27 per strain per age, both sexes included. The *p*-values obtained for each two-group comparison from Dunnett’s multiple comparison test are listed in Supplementary table ST3.

To determine the nature and progression of axial changes, AL was measured over a period of one year and compared to age-matched B6 controls (Fig. 3). Mixed-model ANOVA revealed significant main and interaction effects of mouse strain and age (strain: F(4,101) = 98.45; age: F(2.809, 200.4) = 1762; and interaction: F(12, 214) = 4.469; *P <* 0.0001), and Dunnett’s multiple comparisons test identified significant effects of individual strains (adjusted *P* values for strains at all ages are listed in Supplementary Table ST3). AL increased progressively with age in all strains of mice, but individual mouse strains differ in the extent of their axial growth with age (Fig. 3). The *Prss56*^*glcr4*^, *Mfrp*^*rd6*^, and *Adipor1*^*tm1Dgen*^ mutants had a significantly shorter AL relative to B6 controls at all time points studied, with an exception for *Adipor1*^*tm1Dgen*^ at one month of age. At 1 and 4 months of age, AL shortening in *Prss56*^*em2(IMPC)J*^ mice was indistinguishable from that of *Prss56*^*glcr4*^ mice, so further time points were not examined (Supplementary Fig. S2). Compared to B6 controls, *C1qtnf5*^*tm1.1(KOMP)Vlcg*^ mice did not exhibit changes to AL except at 12 months of age, where a small but statistically significant increase was observed. Together, these observations indicate microphthalmia in all models examined except *C1qtnf5*^*tm1.1(KOMP)Vlcg*^ mice.

### 3.4. Effect of sex and body weight on AL

To provide a more robust statistical analysis, we accounted for possible effects of sex (Niu et al., 2016; Richter et al., 2017; Roy et al., 2015) and body weight. Factorial ANOVA of AL data confirmed statistically significant main and interaction effects of strain, age and sex (Table 2). The effect of sex may be due in part to the larger body size of males. To account for this, we performed ANCOVA and included body weight (as a measure for body size) as a covariate (Table 2). ANCOVA provided a slightly better fit than ANOVA (ANCOVA: adj *R*^*2*^_=_ 0.952, RMSE = 0.037 and F Ratio = 169.0752 (Prob *>* F =_*<*_0.0001); ANOVA: adj *R*^*2*^_=_ 0.949, RMSE = 0.039 and F Ratio = 162.5134 (Prob *>* F = *<*0.0001)). After controlling for body weight, a small (roughly 1 %) but significant effect of sex on AL was observed in several strains. Importantly, the interaction terms involving sex were no longer significant after adjusting for body weight, indicating that sex-based AL variations do not influence the AL differences due to strain and/or age. This analysis highlights the importance of accounting for both body size and sex when interpreting data for differences due to strain effects. Importantly, the trends observed in our initial analysis remained significant even after this more robust statistical treatment.

### 3.5. Analysis of anterior and posterior axial measures

**Fig. 4.**
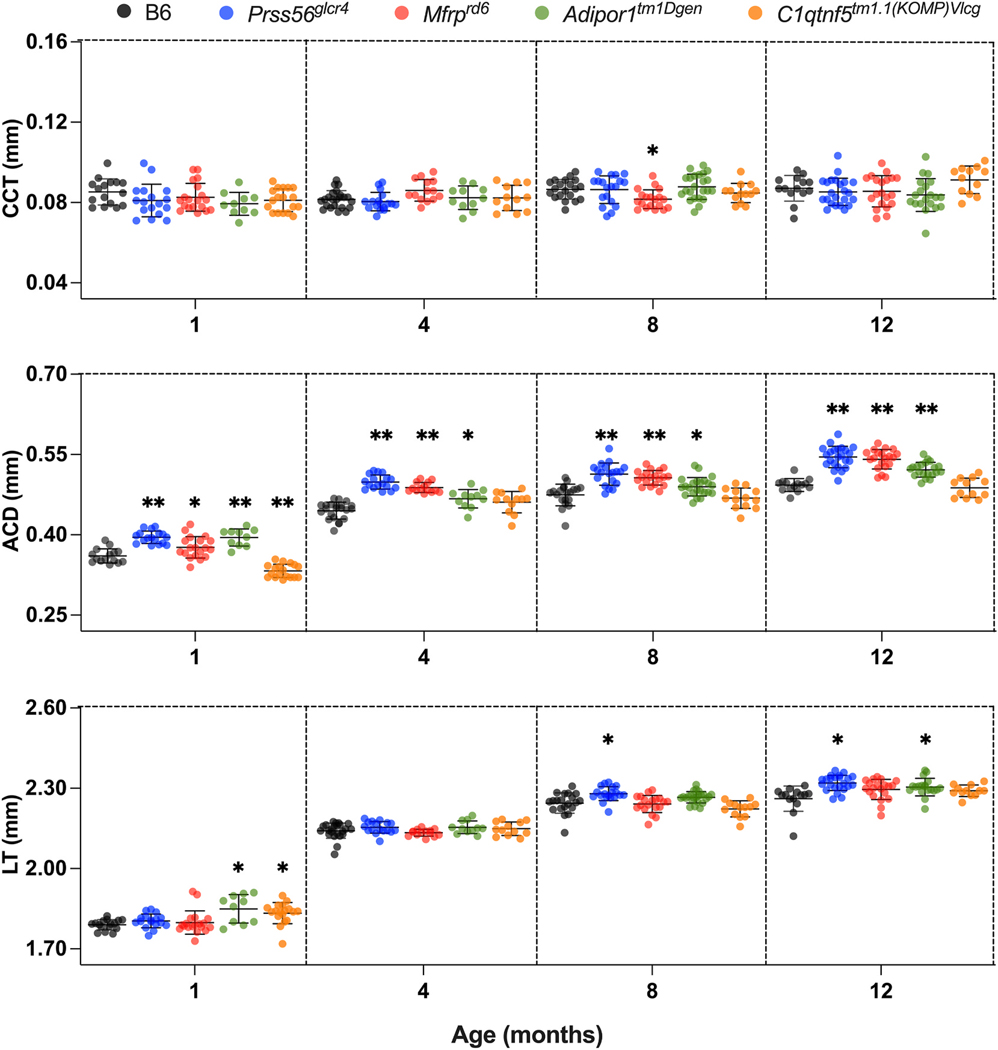
Comparative analysis of ocular parameters in the anterior of the eye, for B6 control and PMN models at 1, 4, 8, and 12 months of age. B6, *Prss56*^*glcr4*^, *Mfrp*^*rd6*^, *Adipor1*^*tm1Dgen*^, and *C1qtnf5*^*tm1.1(KOMP)Vlcg*^ mice are represented in gray, blue, red, green and orange circles, respectively. Statistical details are the same as in Fig. 3, with Dunnett’s test values listed in Supplementary Table ST5–ST7.

**Fig. 5.**
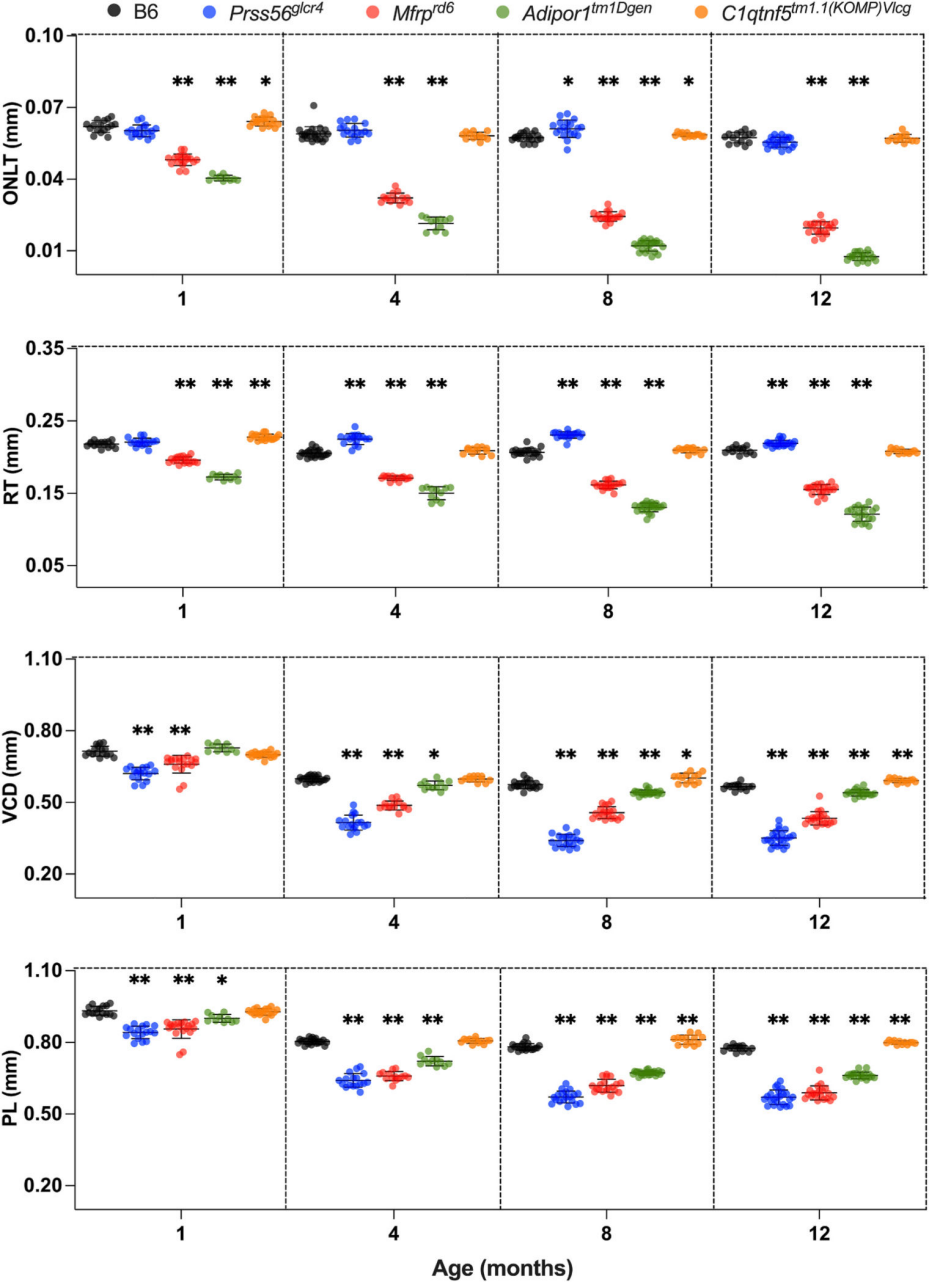
Comparative analysis of ocular parameters in the posterior eye, for B6 control and PMN models at 1, 4, 8, and 12 months of age. B6, *Prss56*^*glcr4*^, *Mfrp*^*rd6*^, *Adipor1*^*tm1Dgen*^, and *C1qtnf5*^*tm1.1(KOMP)Vlcg*^ mice are represented with gray, blue, red, green, and orange circles, respectively. Statistical details are the same as in Fig. 3, with Dunnett’s test values listed in Supplementary Table ST8–ST11.

To determine which ocular tissues contributed to the observed changes in AL, we evaluated axial (CCT, ACD, LT, VCD, PL) and retinal (ONLT, RT) parameters (shown in Figs. 4 and 5) using mixed-model ANOVA followed by post-hoc analysis (Supplementary tables ST4–ST10). Central corneal thickness (CCT) did not differ significantly between mutants and control (Fig. 4). Lens thickness (LT) increased with age in all strains and was slightly increased in *Prss56*^*glcr4*^ and *Adipor1*^*tm1Dgen*^ mice compared to B6 controls at 8 and 12 months of age. Anterior chamber depth (ACD) increased in all strains with age but varied such that all mutant strains, except *C1qtnf5*^*tm1.1(KOMP)Vlcg*^, had longer ACD than B6 controls.

PL and VCD decreased with age in all mice studied but to a greater degree in the mutants than controls (Fig. 5). ONLT and RT decreased significantly in *Mfrp*^*rd6*^ and *Adipor1*^*tm1Dgen*^ mice, but not in *Prss56*^*glcr4*^ mice, which exhibited increased ONLT and RT when compared to B6. *C1qtnf5*^*tm1.1(KOMP)Vlcg*^ mice exhibited an increased PL, VCD, and RT compared to controls. The association between the change in AL and change in different axial and retinal biometric parameters was confirmed by a significant positive correlation of AL with ACD and LT, and negative correlation of AL with VCD and PL for all of the strains (Supplementary Fig. S3 and Table ST11). The slope obtained from the linear regression fit differed such that all mutants except *C1qtnf5*^*tm1.1 (KOMP)Vlcg*^ had a steeper slope than controls for PL and VCD, representing a greater change in PL and VCD in mutants than control as mice age. *Mfrp*^*rd6*^ and *Adipor1*^*tm1Dgen*^ showed a significantly steeper slope between change in AL and change in ONLT and RT in aging mice, as compared to other strains confirming a faster decrease in ONLT and RT. *Prss56*^*glcr4*^ showed a positive correlation between AL and RT changes, with RT increasing with an increase in AL.

To visualize which tissues contribute substantially to ocular growth, we replotted the ACD, LT, PL, and AL data described above, subtracting the value of each of these parameters in B6 mice at one month of age from all values. The resulting plot (Supplementary Fig. S4) emphasizes the change in each parameter with age. This analysis shows that lens growth dominates the increase in ocular size with age. A defect in posterior growth was evident from the progressive decrease in PL with age in *Prss56*, *Mfrp*, and *Adipor1* mutants. By contrast, posterior growth was unaffected in *C1qtnf5* mutant mice, while an increase in LT and a decrease in ACD was apparent in the youngest *C1qtnf5* mutant mice examined.

### 3.6. Analysis of corneal radius of curvature

**Fig. 6.**
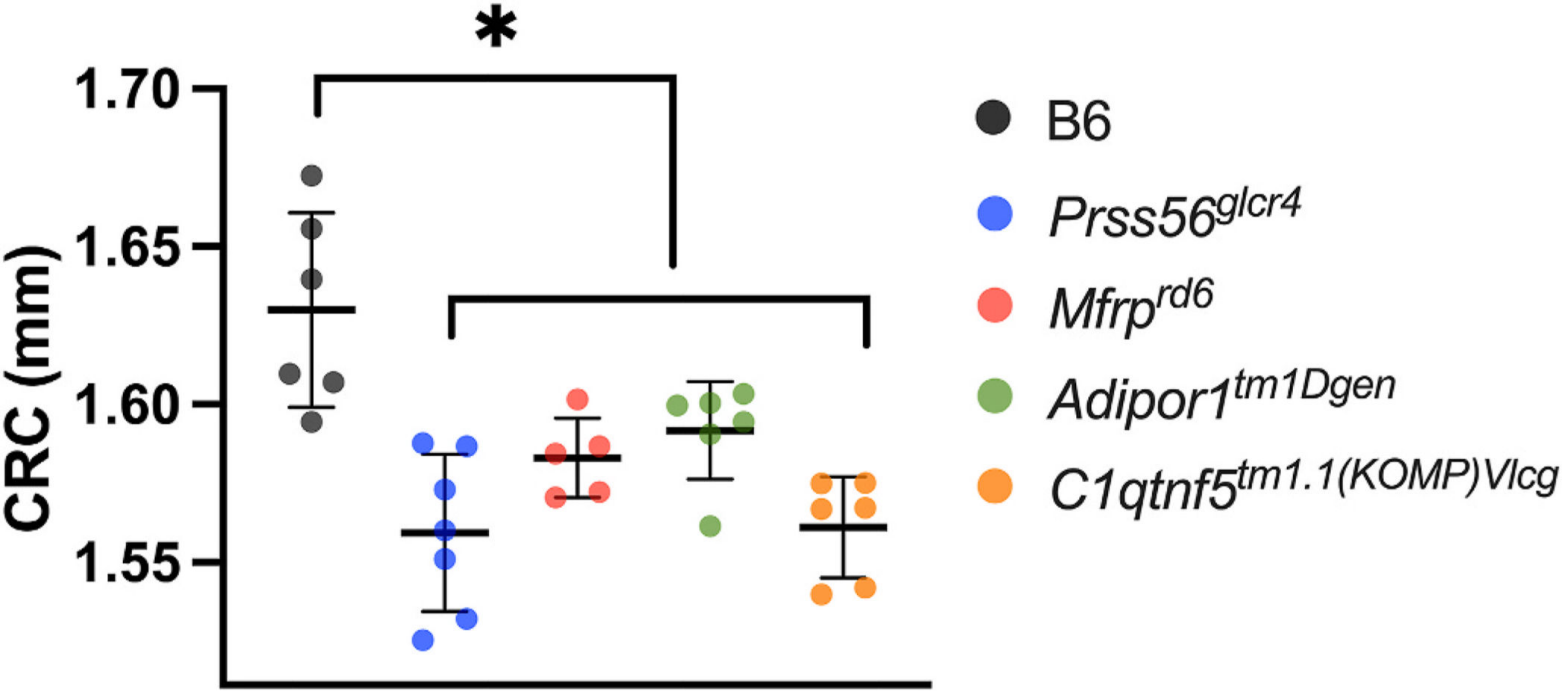
Comparative analysis of CRC in B6 and mutant mice at 4 months of age. B6, *Prss56*^*glcr4*^, *Mfrp*^*rd6*^, *Adipor1*^*tm1Dgen*^, and *C1qtnf5*^*tm1.1(KOMP)Vlcg*^ mice are represented in gray, blue, red, green and orange circles, respectively. The CRC differed significantly between B6 and all mutant strains, as indicated with asterisk (*) above the mutants. *p < 0.05. n = 5–6 per strain, both sexes included. The *p*-values obtained for comparison between B6 and mutants from the ANOVA, followed by Dunnett’s multiple comparison test are listed in Supplementary Table ST13. Mean ± SD values are indicated.

Corneal radius of curvature (CRC) was assessed in 4-month-old mutant and control B6 mice, and compared using ANOVA, followed by post-hoc analysis (Fig. 6 and Supplementary Table ST12). Notably, CRC differed significantly in all the mutants; all the mutants had significantly shorter CRC than B6 mice (Fig. 6). To examine sex effects on CRC, factorial ANOVA was performed to determine individual and interaction effects of strain and sex. Significant effect of strain (F Ratio = 9.4521, Prob *>* F = 0.0002) was observed whereas no significant effect of sex (F Ratio = 0.1511, Prob *>* F = 0.7016) or their interaction (Strain * Sex: F Ratio = 0.6956, Prob *>* F = 0.6038) was seen, confirming that the difference observed in CRC were mainly due to strain. Mean values for the measurements used in this study are provided in Supplementary tables ST13 and ST14, respectively.

### 3.7. Timecourse of fundus pathology

**Fig. 7.**
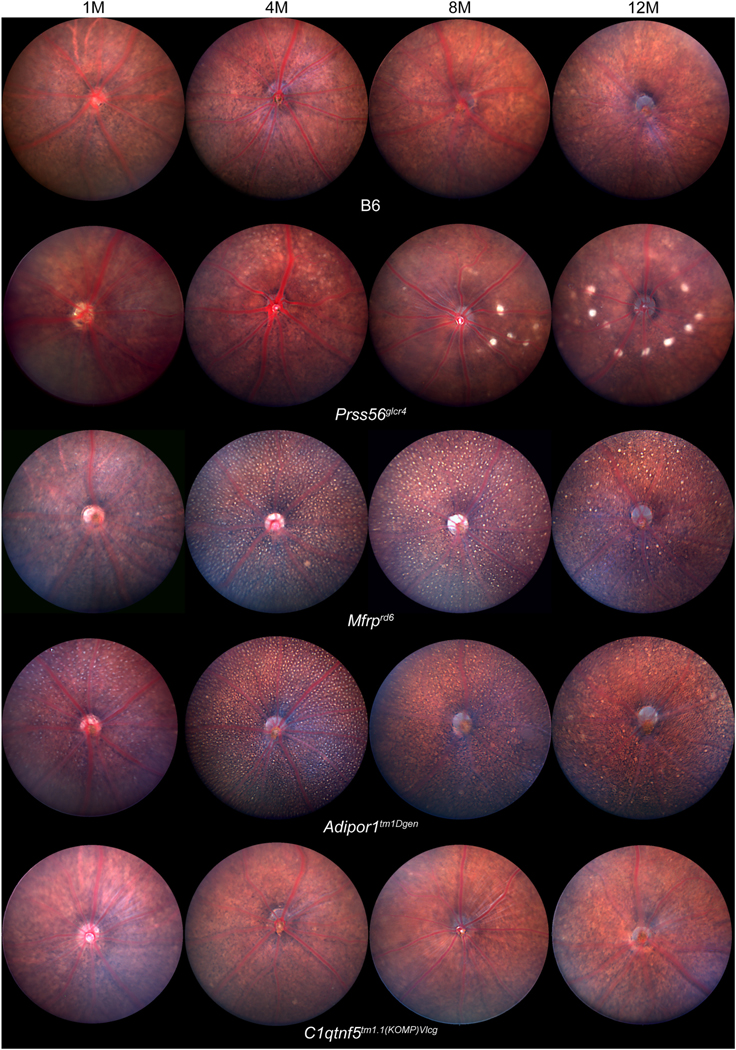
Fundus photographs of *Prss56*^*glcr4*^, *Mfrp*^*rd6*^, *Adipor1*^*tm1Dgen*^, and *C1qtnf5*^*tm1.1(KOMP)Vlcg*^ mice and B6 controls, at 1, 4, 8, and 12 months of age, showing a progressive change in fundus appearance with age. *Prss56*^*glcr4*^ mice develop large bright central fundus spots surrounding the optic nerve. *Mfrp*^*rd6*^ and *Adipor1*^*tm1Dgen*^ mutants develop uniformly sized small spots over the full fundus that are similar in appearance and distribution. These spots are less abundant in older mice. *C1qtnf5*^*tm1.1(KOMP)Vlcg*^ mice do not develop spots and appear similar to B6 controls at all ages studied (n = 6–10, both sexes included for each strain).

**Fig. 8.**
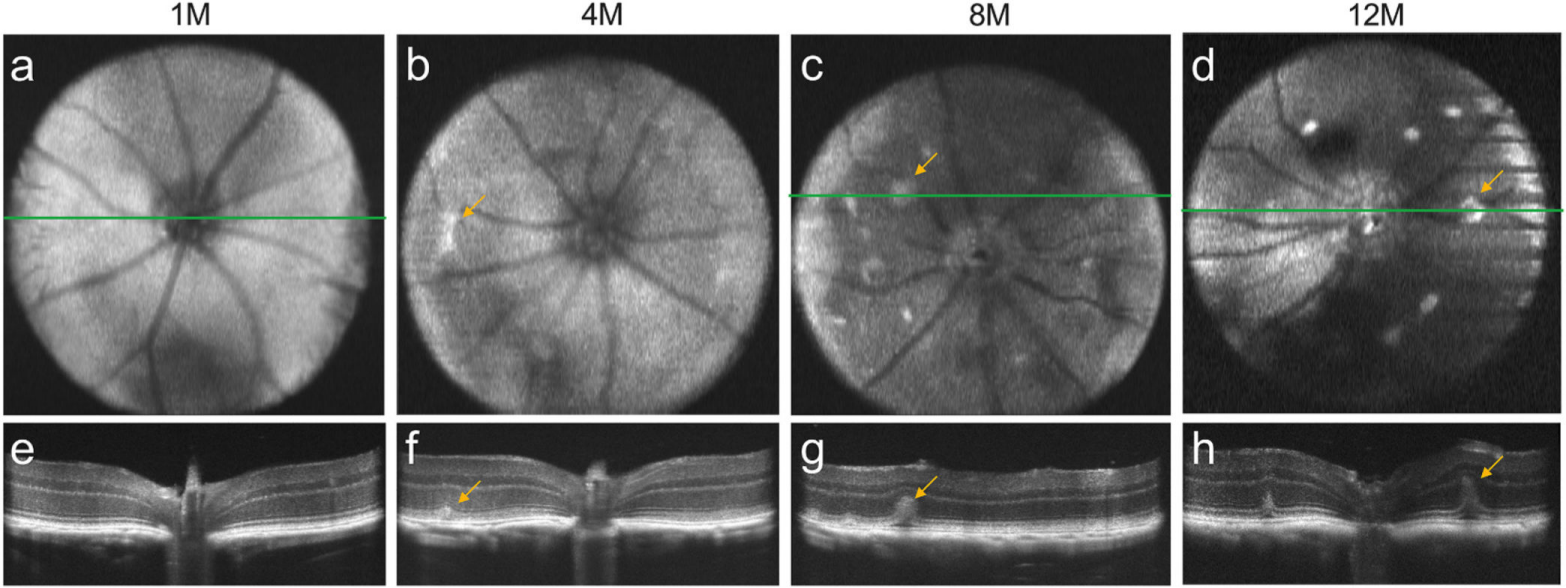
Assessment of retinal fold development in *Prss56*^*glcr4*^ mice by *in vivo* imaging. **a-d**. SD-OCT *en face* images. E-H. Corresponding B-scans showing progressive development of retinal folds in the posterior eye of mice *Prss56*^*glcr4*^ mice at 1 (**a**, **e**), 4 (**b**, **f**), 8 (**c**, **g**), and 12 (**d**, **h**) months of age. A green line in the *en face* image depicts the region that corresponds to the respective B-scan image. Yellow arrows highlight atypical spots in the *en face* view and corresponding retinal folds observed in the B-scan images. Representative images from different mice are shown (n = 6 at each age).

*Mfrp*^*rd6*^ and *Adipor1*^*tm1Dgen*^ mice share multiple disease phenotypes, including an early development of fundus spots (Gogna et al., 2022; Hawes et al., 2000; Kameya et al., 2002; Kautzmann et al., 2020; Rice et al., 2015). To determine disease progression, fundus photo-documentation was carried out at 1, 4, 8, and 12 months of age (Fig. 7). At one month of age, uniformly sized, white fundus spots were detected in both *Mfrp*^*rd6*^ and *Adipor1*^*tm1Dgen*^ mice, and were distributed throughout the fundus by four months of age. In *Prss56*^*glcr4*^ mice aged eight months and older, large, bright spots were distributed along the central fundus and surrounding the optic nerve. Fundus phenotypes in *Prss56*^*em2(IMPC)J*^ mice emulated those observed in *Prss56*^*glcr4*^ mice (Supplementary Fig. S2). Interestingly, retinal folds were observed by OCT, corresponding to the fundus spots seen in *Prss56*^*glcr4*^ mice (Supplementary Fig. S5), which progressively increased in size and number as the animals aged (Fig. 8). *C1qtnf5*^*tm1.1(KOMP)Vlcg*^ mutants had a normal fundus appearance throughout the 12-month timecourse.

### 3.8. Histological and immunohistochemical analysis

To further test for shared pathological features among PMN mutants, we examined B6 and mutant strains by histological analysis of ocular sections at 1 and 12 months of age (Supplementary Fig. S6). In comparison, both methods presented similar changes in ONLT, where a decreased ONLT seen by OCT corresponded to PR loss seen by histological analysis, validating the use of OCT for measurement of ocular biometry.

We also immunostained sections for GFAP expression in Müller glia cells, known to be activated in response to retinal degeneration (Fernandez-Sanchez et al., 2015) and retinal laser injury (Tackenberg et al., 2009) (Supplementary Fig. S7). Our results show Müller glial activation in 4-month-old *Mfrp*^*rd6*^ and *Adipor1*^*tm1Dgen*^ mutants as depicted by strong GFAP staining of Müller cell processes. There was no evidence of Müller glial activation in 4-month-old *Prss56*^*glcr4*^ and *C1qtnf5*^*tm1.1(KOMP)Vlcg*^ mice.

## 4. Discussion

In this study, we examined ocular growth in three known and two prospective mouse PMN models using OCT biometry at 1, 4, 8, and 12 months of age. Retinal phenotypes were also explored by fundus imaging, OCT, and histological analyses. Our results underscore the use of *Prss56*, *Mfrp*, and *Adipor1* mutants as related models for studying PMN. The results also identify *C1qtnf5* as a gene that contributes to anterior ocular growth.

### 4.1. Axial ocular biometry as a function of age

Axial biometric parameters obtained from the eye at regular time-points between 1 and 12 months of age revealed a prominent contribution of lens growth to the postnal ocular growth of all strains examined. In all strains, we observed a rapid ocular growth (8–12 % increase in AL from 1 to 4 months of age) followed by slower growth (4–5 % increase in AL from 4 to 12 months of age), consistent with the two-phase growth pattern reported previously (Chou et al., 2011; Puk et al., 2006; Schmucker and Schaeffel, 2004; Yang et al., 2018; Zhou and Williams, 1999; Zhou et al., 2008). In addition, the increase in AL with age was accompanied by an equal or greater increase in LT, an increase in ACD, and a progressive decrease in PL. Similar findings in studies of B6 mice have been reported previously (Chou et al., 2011; Puk et al., 2006; Schmucker and Schaeffel, 2004). In B6 control mice, the age-dependent changes to ACD and PL, which are in opposite directions, effectively cancel each other so that the increase in AL is entirely due to an increase in LT. These changes to ACD and PL reflect a more posterior positioning of the lens with age.

### 4.2. Models with shared PMN phenotypes

Four of the models examined had similar PMN phenotypes. *Prss56*, *Mfrp*, and *Adipor1* mutant strains exhibited a statistically significant 1.4–3.9 % decrease in axial length, similar to values obtained in previous studies (Gogna et al., 2022; Koli et al., 2021; Nair et al., 2011; Paylakhi et al., 2018). The prospective *Prss56* knockout model, characterized here, showed a phenotype similar to that of *Prss56*^*glcr4*^ (Koli et al., 2021; Nair et al., 2011) and an independent knockout model *Prss56*^*Cre*^ (Paylakhi et al., 2018). Although all four models exhibited microphthalmia, AL at all ages differed slightly in the order: *Prss56*^*glcr4*^= *Prss56*^*em2(IMPC)J*^ *< Mfrp*^*rd6*^ < *Adipor1*^*tm1Dgen*^. Indeed, the decrease in the AL of *Adipor1*^*tm1Dgen*^ mice did not become statistically significant until 4 months of age, as reported previously (Gogna et al., 2022). LT and CT were largely unchanged in these models relative to age-matched B6 control mice; significant deviations, where noted, resulted in only a slight increase in LT or decrease in CT. Similar effects on these parameters were observed in *Prss56*^*glcr4*^ and *Mfrp*^*rd6*^ mice (Koli et al., 2021; Nair et al., 2011). ACD was significantly increased compared to B6 at all ages tested; a similar effect in *Prss56*^*glcr4*^ mice was reported previously (Paylakhi et al., 2018). PL varied in the order: *Prss56*^*glcr4*^ = *Prss56*^*em2 (IMPC)J*^ < *Mfrp*^*rd6*^ < *Adipor1*^*tm1Dgen*^ < B6. Thus, the shorter axial length was not due a decrease in CT, LT, or ACD, but rather a decrease in the size of the posterior eye. Finally, the CRC, as determined at 4 months of age, was smaller for all four mutants, indicating a smaller size of the anterior eye. Taken together, these features indicate a shared PMN disease phenotype affecting both anterior and posterior dimensions in the *Prss56*, *Mfrp*, and *Adipor1* mutants studied, with mutant-specific differences in the magnitude of biometric measures.

Biometric parameters of *Mfrp* and *Prss56* mutant strains recapitulate those in human microphthalmia caused by orthologous gene variants, with important exceptions. As in the mouse models, CT and LT are unaffected or slightly increased, while PL, AL, and CRC are significantly reduced in *MFRP* or *PRSS56* posterior microphthalmia (Almoallem et al., 2020; Bacci et al., 2020; Gal et al., 2011; Nair et al., 2011; Nowilaty et al., 2013; Said et al., 2013; Sen et al., 2022; Siggs et al., 2020) and nanophthalmia (Carricondo et al., 2018; Fledelius et al., 2004; Gal et al., 2011; Sundin et al., 2008) [note that some of these studies report corneal power, which is proportional to the inverse of the CRC; increased corneal power corresponds to a decreased CRC]. However, unlike the findings in mouse *Mfrp* and *Prss56* models, ACD is normal or slightly decreased in human *MFRP* or *PRSS56* posterior microphthalmia (Almoallem et al., 2020; Bacci et al., 2020; Gal et al., 2011; Nair et al., 2011; Nowilaty et al., 2013; Said et al., 2013; Sen et al., 2022; Siggs et al., 2020) and is substantially decreased in nanophthalmos (Carricondo et al., 2018; Relhan et al., 2016). It is possible that the difference in the direction of ACD change in mouse and human PMN disease is related to the relatively larger lens in mice (see Introduction), which may cause it to be repositioned more toward the posterior eye in response to a decline in anterior and posterior growth. The relatively large size of the mouse lens may also account for the relatively small decrease in AL (see Introduction). In summary, the *Mfrp* and *Prss56* models of PMN faithfully replicate the human disease, except for a relatively smaller effect on AL and an increase in ACD, which may result from the relatively larger lens in mice.

### 4.3. C1qtnf5 influences anterior ocular growth

We examined a *C1qtnf5* knockout strain because previous studies suggested *Mfrp* and *C1qtnf5* might function in shared pathways (Chavali et al., 2010; Kameya et al., 2002; Mandal et al., 2006; Shu et al., 2006). Compared to age-matched B6 controls, *C1qtnf5*^*tm1.1(KOMP)Vlcg*^ mice did not show a significantly shorter AL, indicating that the mutation does not result in PMN. Nevertheless, ACD was decreased and LT was increased at 1 month of age, and CRC was reduced at 4 months of age, while AL remained similar to that of B6 controls. These results raise the possibility that *C1qtnf5* influences the growth of the lens and shape of the anterior eye. Interestingly, *C1QTNF5* missense variants in late-onset retinal degeneration patients are associated with a lengthening of anterior zonules (Ayyagari et al., 2005; Subrayan et al., 2005), which comprise the lens suspensory apparatus. It therefore has been suggested that *C1QTNF5* plays a role in zonule production or maintenance. Thus, it is possible that lens zonules are altered in *C1qtnf5* knockout mice, leading to a change in lens position. Unexpectedly, the changes to ACD and LT in *C1qtnf5*^*tm1.1(KOMP)Vlcg*^ mice were transient, as significant differences were not observed in older animals. Of interest, the CRC at 4 months of age was significantly smaller compared to controls, as in the other mutants examined, while AL matched that of B6 controls. Taken together, these findings suggest a possible influence of *C1qtnf5* on anterior ocular growth.

### 4.4. Retinal degeneration

In addition to assessing changes in axial measures, our studies explored whether retinal phenotypes were shared among the PMN models. To assess retinal and photoreceptor degeneration, we measured RT and ONLT in OCT B-scans and identified qualitative retinal changes by histological analysis. A small but statistically significant decline in B6 RT and ONLT was observed during the 12 months assessed by OCT, similar to the 5–15 % retinal thinning reported for B6 mice that were *>*24 months of age (Ferdous et al., 2021; Samuel et al., 2011). These biometric parameters may decline with age in part due to the thinning of posterior tissues as the globe increases in size. A more rapid decline in RT and ONLT was detected in *Mfrp*^*rd6*^ and *Adipor1*^*tm1Dgen*^ mice with age, indicating progressive photoreceptor degeneration, as found in previous reports (Gogna et al., 2022; Hawes et al., 2000; Kameya et al., 2002; Kautzmann et al., 2020; Rice et al., 2015). Immunohistochemical evidence of GFAP upregulation, a signature of Müller glial cell activation (Fernandez-Sanchez et al., 2015), provided further support for retinal degeneration in these models. By contrast, compared to B6 controls, RT and ONLT were higher at all ages in *Prss56*^*glcr4*^ mice, as reported previously (Paylakhi et al., 2018), and in *Prss56*^*em2(IMPC)J*^ mice. The increase in RT and ONLT, and consequent decrease in VCD, may be due to the compression of the retinal tissue in a smaller ocular globe as postulated in *MFRP* and *PRSS56* posterior microphthalmia (Almoallem et al., 2020; Boynton and Purnell, 1975; Gal et al., 2011; Nair et al., 2011; Sen et al., 2022). There was no evidence of glial activation in *Prss56*^*glcr4*^ and *C1qtnf5*^*tm1.1(KOMP)Vlcg*^ mice, consistent with a lack of apparent retinal degeneration in these models. Thus, retinal degeneration or thickening is not a shared feature of the PMN models.

VCD differences between the mutants and B6 controls reflect changes in RT and/or PL. As we have already considered the effects of mutations on PL, we have not reviewed the effects on VCD. However, we include VCD as a reference for others who use this parameter.

### 4.5. Retinal spots

Shared posterior ocular abnormalities were detected by color fundus imaging, OCT, and histology. Fundus imaging of *Mfrp*^*rd6*^ and *Adipor1*^*tm1Dgen*^ mice revealed uniform and evenly distributed white spots across the fundus by 1 month of age, typical of the white flecks observed in humans with *MFRP* mutations and flecked retinal disorder (Hawes et al., 2000; Kameya et al., 2002). These spots became most prominent at 4 months of age and slowly dissipate thereafter. In these and related *Mfrp* and *Adipor1* mutants, the white spots correspond to activated immune cells, including macrophages and microglia (Fogerty and Besharse, 2011; Hawes et al., 2000; Lewandowski et al., 2022; Rice et al., 2015), which often accumulate sub-retinally in response to photoreceptor cell degeneration (Almoallem et al., 2020; Langmann, 2007). Accordingly, these small white spots were not observed in *Prss56* or *C1qtnf5* mutant strains, which do not exhibit retinal degeneration.

Fundus images of *Prss56*^*glcr4*^ mice at 4 months of age and older presented larger and brighter spots than those observed in *Mfrp* and *Adipor1* mutants, predominantly near the optic nerve head. These spots corresponded to retinal folds observed by OCT and histology, and increased in both size and number with age. The *Prss56*^*glcr4*^ retinal folds may be analogous to papillomacular folds associated with human *PRSS56* mutations (Almoallem et al., 2020). Like the thickened retina discussed above, these folds are thought to result from normal retinal development in the context of arrested scleral growth (Almoallem et al., 2020; Gal et al., 2011; Nair et al., 2011; Sen et al., 2022), which may also explain their formation in *Prss56* mutant mice. Interestingly, spots similar to those in *Prss56*^*glcr4*^ mice were observed in mouse *Tmem98* mutants (Cross et al., 2019; Hassall et al., 2023), raising the possibility that the *Tmem98* mutant mice might also be microphthalmic. Although an abnormal AL phenotype was not reported in those studies, a statistically nonsignificant AL decrease (7 %) was observed in one *Tmem98* mutant (Cross et al., 2019), which might reveal an effect if characterized further.

Throughout the 12-month timecourse, *C1qtnf5*^*tm1.1(KOMP)Vlcg*^ mice displayed a normal fundus appearance. These findings contrast with previous reports that show a fundus spotting phenotype in the *C1qtnf5* S163R knock-in model (*C1qtnf5*^*tm1.1Itl*^) of late-onset retinal degeneration (Chavali et al., 2011). The abnormal fundus phenotype of heterozygous *C1qtnf5* S163R knock-in mice was observed at 20–21 months of age (Chavali et al., 2011), a time point not assessed in our model. *C1qtnf5*^*tm1.1Itl*^ is likely a gain-of-function allele, while *C1qtnf5*^*tm1.1(KOMP) Vlcg*^ is a null allele, as confirmed by the lack of expression as assessed by qRT-PCR. Thus, although a late fundus phenotype may yet be observed in *C1qtnf5*^*tm1.1(KOMP)Vlcg*^ mice, allelic differences may account in part for the phenotypic disparities.

### 4.6. Relevance to gene networks regulating ocular size

Our studies provide new insights into a network of genes associated with ocular growth. As mentioned, *BEST1, CRB1, MFRP, MYRF, PRSS56,* and *TMEM98* variants are uniquely associated with the PMN subtype of human microphthalmic disease, raising the possibility that these genes contribute to a shared genetic network regulating ocular growth. Several studies have provided evidence for interactions among these genes. The loss of *Mfrp* expression among *Mfrp* mutant lines results in increased *Prss56* transcripts (Koli et al., 2021; Soundararajan et al., 2014). *Mfrp* and *C1qtnf5* are expressed from a dicistronic transcript (Hayward et al., 2003; Kameya et al., 2002) and the encoded proteins interact (Mandal et al., 2006; Shu et al., 2006). *Mfrp*^*rd6*^ mice have decreased levels of ADIPOR1 in the RPE layer, suggesting a potential interaction (Sluch et al., 2018). Genetic interaction of *Mfrp* and *Adipor1* has been observed in retinal degeneration and fundus spotting, although these genes do not appear to interact with respect to AL (Gogna et al., 2022). *Tmem98* transcription in the RPE is reduced in *Myrf* conditional knockout mice, suggesting interaction of these genes (Garnai et al., 2019). Furthermore, TMEM98 interacts with MYRF to regulate autoproteolytic production of an MYRF cytosolic domain that functions as a transcription factor. Our studies identifying *C1qtnf5* as a regulator of anterior ocular growth raises the possibility that this gene may participate in this network. Indeed, recent evidence indicates that C1QTNF5 functions as a ligand of ADIPOR1 (Miyagishima et al., 2021). Future studies are needed to extend these genetic networks and to identify the molecular pathways in which the encoded proteins function.

### 4.7. Limitations

This study has several limitations. Our calculations of axial dimensions used accepted refractive index values for each ocular segment but did not account for the potential variation of these values with age and strain, which may confound the measurement of axial distances. Further, axial measures do not provide three-dimensional information about the size of the eye and may be misleading as a measure of ocular size if offset by changes in ocular shape. Noninvasive techniques that are insensitive to refractive index, such as magnetic resonance imaging (Tkatchenko et al., 2010), may resolve this concern. Another limitation is the use of a *C1qtnf5* knockout mutant that retains a *lacZ* reporter cassette within the *C1qtnf5* gene. Targeting constructs that retain *lacZ* may result in unintended effects on local gene transcription (Peck et al., 2021) and even neuronal toxicity (West et al., 2016). It may be informative to perform ocular biometry in an independent *C1qtnf5* knockout strain (Borooah et al., 2023). Finally, as the mutant alleles targeted in this study were introgressed for five generations onto B6, the tested strains are not fully congenic and retain an estimated 1.6 % of the donor strain genome in unlinked regions. This issue may be of particular relevance to the *C1qtnf5* and *Prss56* knockout strains, which were derived from C57BL/6N mice that differ from B6 mice at multiple loci (Simon et al., 2013), and may be resolved by further backcrosess. Littermate controls may help to minimize potential confounds due to variations in strain genetic background.

### 4.8. Summary

In summary, this study provided a detailed comparison of changes in ocular axial tissue thicknesses, chamber depths, and corneal radius of curvature as a function of age, confirming an overlapping microphthalmia phenotype in three known mutant strains and a fourth prospective strain. In addition, studies of a novel *C1qtnf5* knockout mutant strain suggested that *C1qtnf5* plays a role in anterior ocular growth. Altogether, these mouse models may help to elaborate the molecular networks that regulate ocular growth.

## Supplementary Material

Figure S1 - Figure S7 Table ST1-ST12

Tables ST13 & ST14

Appendix A. Supplementary data

Supplementary data to this article can be found online at https://doi.org/10.1016/j.exer.2025.110335.

## Figures and Tables

**Table 1:** Known and prospective models of PMN studied in this paper. All mutant alleles were generated or introgressed onto the B6 background for at least five generations. nd, not determined.

Gene	Allele	Mutation	RNA	Protein
*Adipor1*	*tm1Dgen*	Targeted deletion of c.107–198 in exon 2 (Bjursell et al., 2007)	Reduced expression ( Sluch et al., 2018)	Loss of protein (Bjursell et al., 2007; Sluch et al., 2018)
*C1qtnf5*	*tm1.1 (KOMP)* *Vlcg*	Reporter-tagged deletion allele (1090 bp, part of exons 2 and 3) (MGI) (Dickinson et al., 2016; Lloyd, 2011)	Reduced expression (this paper)	nd
*Mfrp*	*rd6*	4-bp deletion in intron 4 splice donor sequence resulting in a cryptic splice site causing in-frame skipping of exon 4 (Kameya et al., 2002)	Increased expression at P28 ( Won et al., 2008)	Loss of protein (Won et al., 2008)
*Prss56*	*glcr4*	T to A transversion in exon 11 splice donor sequence resulting in transcription of an additional 54 bp in intron 11 leading to a premature stop codon (Nair et al., 2011)	Increased expression (Nair et al., 2011)	nd
*Prss56*	*em2 (IMPC)J*	Cas9-guided excision of 845 bp sequence removing exons 3 and 4 and 601 bp of flanking intron predicted to result in early termination due to loss of splice acceptor/donor (MGI) (Dickinson et al., 2016; Lloyd, 2011)	Increased expression (this paper)	nd

**Table 2:** Effect tests showing main and interaction effects of strain, age, and sex on AL, with and without controlling for body weight covariate. After adjusting for covariate, the F Ratio for the sex term decreases and interaction terms involving sex become insignificant ([Prob > F] > 0.01). The covariate significantly adjusted the dependent variable AL as determined from significant F Ratio ([Prob > F] < 0.01; highlighted in bold).

Factorial ANOVA			Factorial ANOVA		
Source	F Ratio	Prob > F	Source	F Ratio	Prob > F
Strain	120.2730	**<0.0001**	Strain	116.3199	**<0.0001**
Age	1644.1188	**<0.0001**	Age	428.2915	**<0.0001**
Strain*Age	5.2722	**<0.0001**	Strain*Age	5.7184	**<0.0001**
Sex	52.3704	**<0.0001**	Sex	12.7802	**0.0004**
Strain*Sex	3.5291	**0.0078**	Strain*Sex	2.9455	0.0206
Age*Sex	2.0947	0.1010	Age*Sex	2.2157	0.0864
Strain*Age*Sex	0.9591	0.4882	Strain*Age*Sex	0.6914	0.7596
			Covariate (body weight)	20.1007	**<0.0001**

## Data Availability

Data will be made accessible in the Mendeley Data Repository. A biometric survey of known and prospective murine models of posterior microphthalmia-nanophthalmia. Gogna et al. (Original data) (Mendeley Data).
