# Supplementary material for: A biometric survey of known and prospective murine models of posterior microphthalmia-nanophthalmia
Source: Exp Eye Res. Author manuscript; Available in PMC 2026 Feb 16. (PMC12906678; doi:10.1016/j.exer.2025.110335)
Supplement: Figure S1 - Figure S7 Table ST1-ST12 [file NIHMS2119121-supplement-SupplementaryS1-S7_ST1-ST12.pdf]

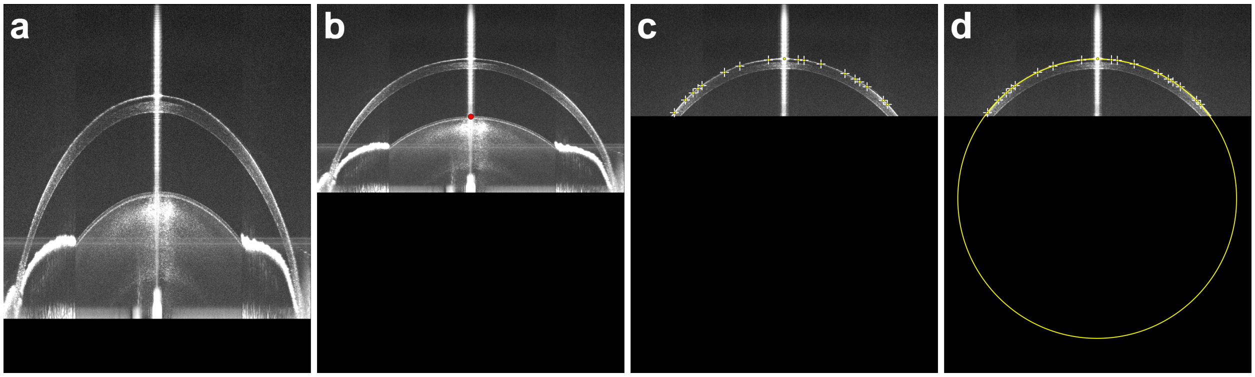

Figure S1: Calculation of the corneal radius of curvature (CRC) using a macro in Fiji. **a**. Registering and averaging a multi-frame OCT B-scan of the anterior segment of the mouse eye, obtained using a telecentric lens. **b**. Rescaling the image based on distance calibration measurements and manually selecting a point at the apex of the lens (red dot) centered on the Purkinje reflection. **c**. Identifying intensity maxima at the anterior surface of the cornea (yellow dots with crosshairs) within a cropped region of the cornea anterior to the lens. Maxima outside the corneal surface were removed manually. **d**. Fitting a circle to the selected maxima. The CRC is calculated from the radius of this circle based on the pixel-to-mm conversion.

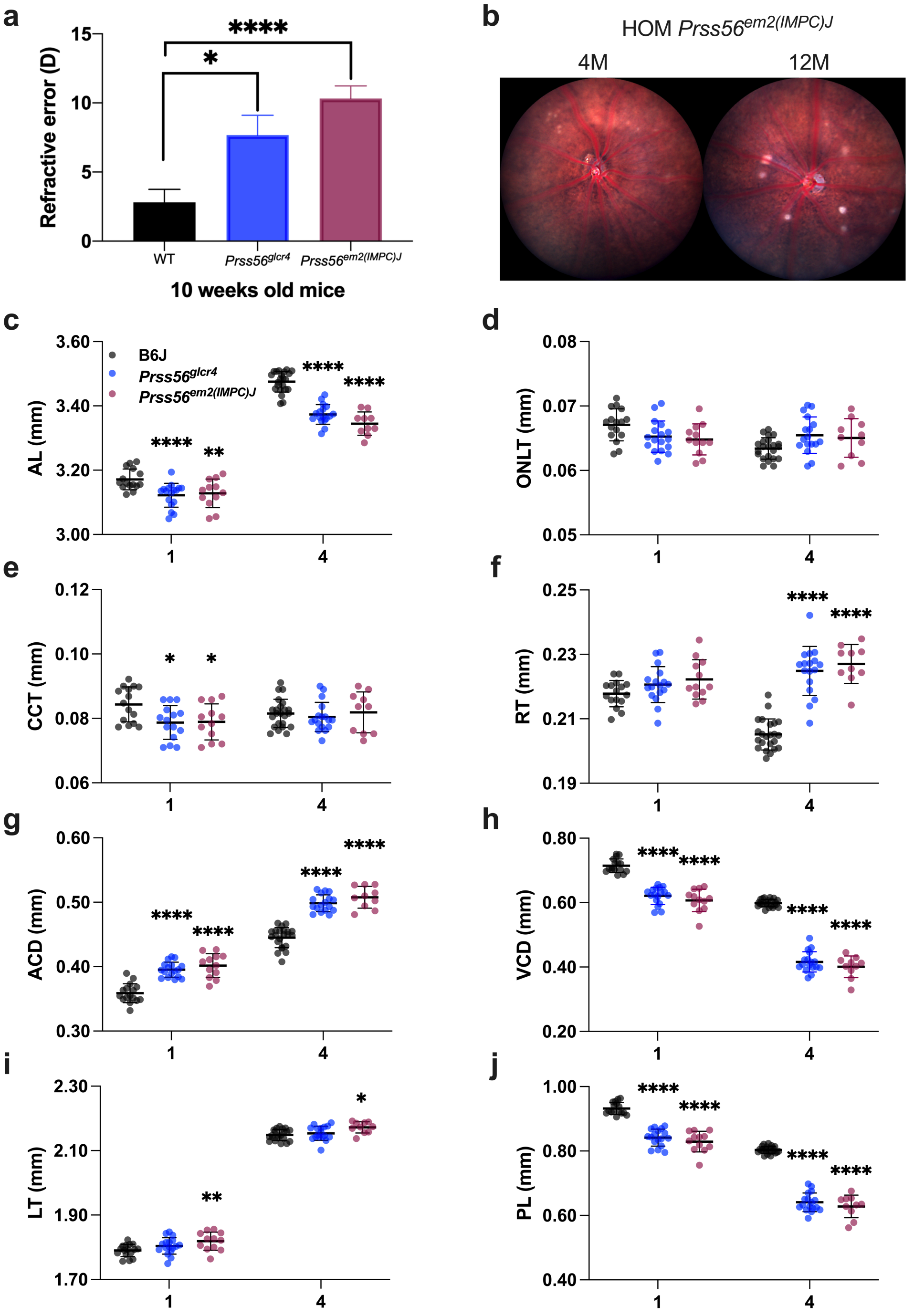

Figure S2: Phenotypic similarities among *Prss56* allelic variants. **a.** Significant changes in refractive error (diopter) observed in *Prss56*^{glcr4} and *Prss56*$^{em2(IMPC)J}$ mice, at 10 weeks of age. **b.** Fundus photos showing spots in *Prss56*$^{em2(IMPC)J}$ homozygotes (HOM), similar to those observed in *Prss56*^{glcr4} homozygotes, at 4 and 12 months (see Figure 7). n = 5 for each age. **c–j.** Comparative analysis shows a similar change in axial and retinal parameters in both *Prss56*^{glcr4} and *Prss56*$^{em2(IMPC)J}$ mice at 1 and 4 months of age, respectively. n = 10–17 mice for each strain, both sexes included. * $p < 0.05$; ** $p < 0.01$; **** $p < 0.0001$.

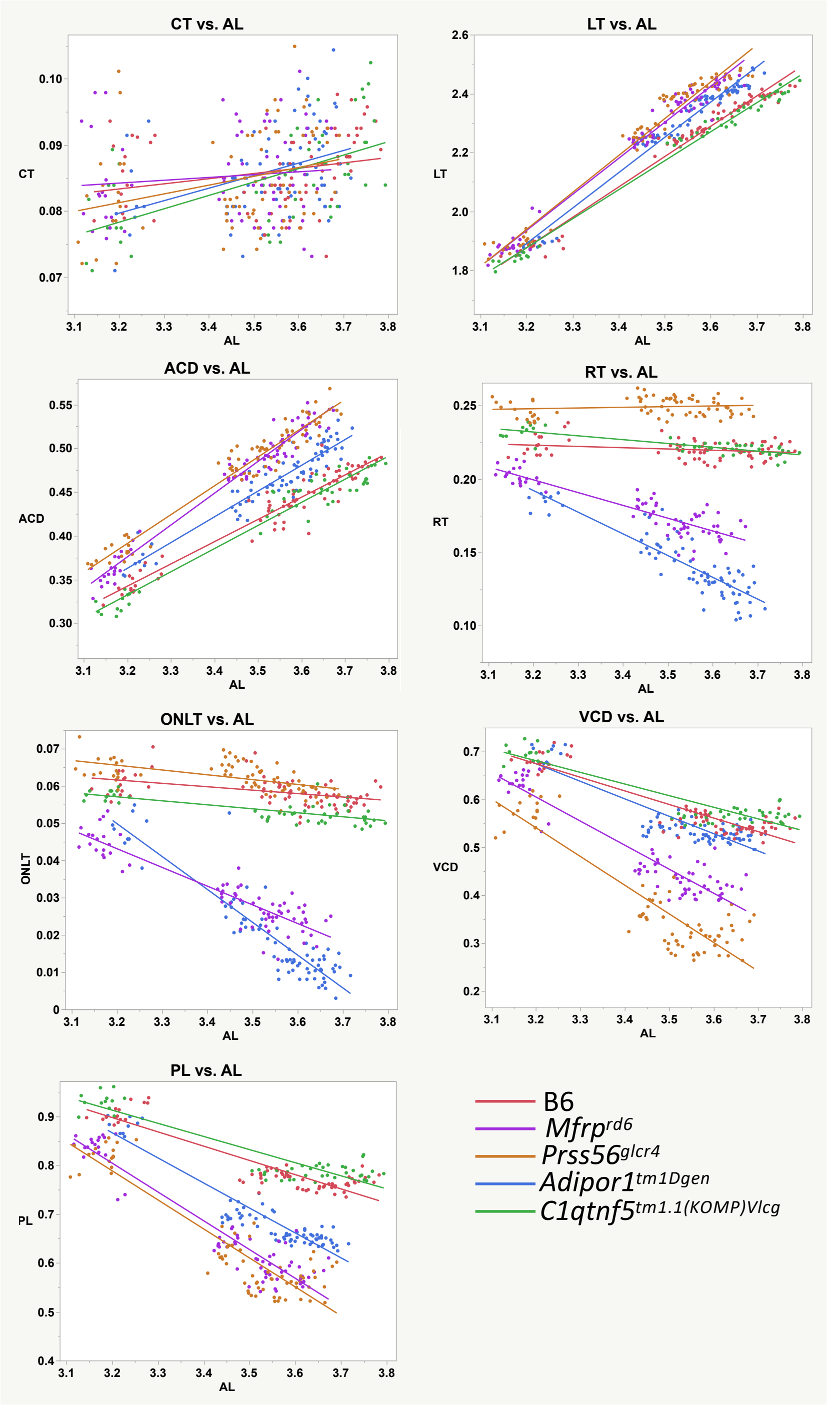

Figure S3: Scatterplots showing the correlation between the change in AL and the change in CT, ACD, LT, ONL, RT, VCD and PL in different mouse strains (B6; red, *Mfrp*^{rd6}; purple, *Prss56*^{glcr4}; orange, *Adipor1*^{tm1Dgen}; blue and *C1qtnf5*$^{tm1.1(KOMP)Vlcg}$; green). The different colored lines represent linear regression fitted to the data points of the individual strains (Regression equation: y = mx + c; where y = CCT/ACD/LT/ONLT/RT/VCD/PL, x = AL, c = intercept and m = slope).

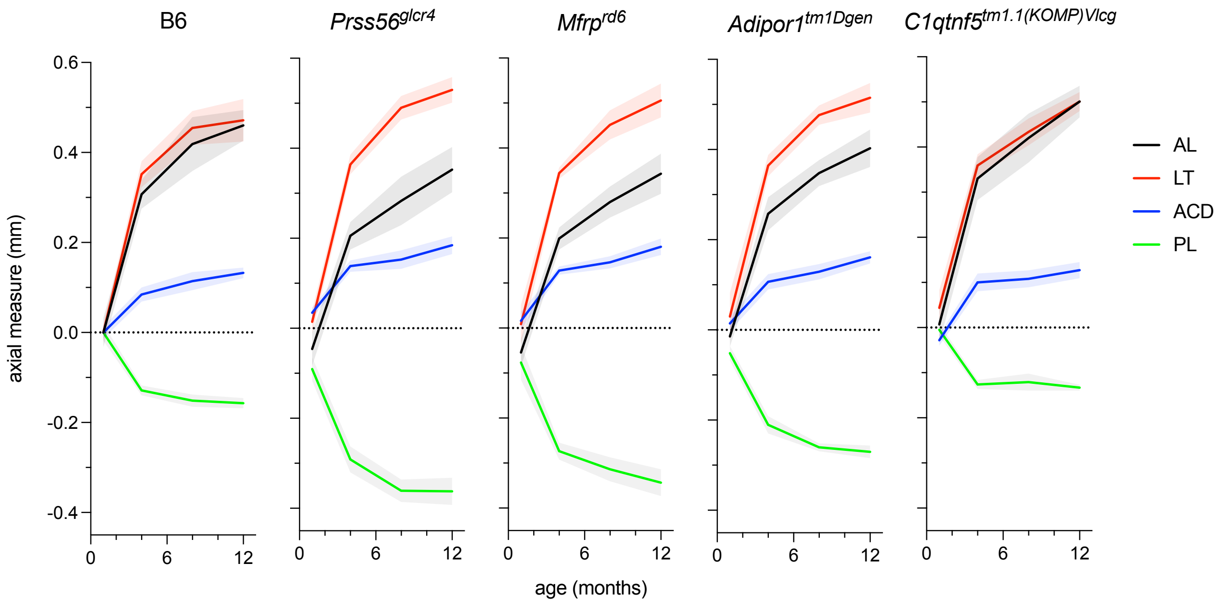

Figure S4: Plot showing change in the individual parameters with age, for each of the mouse strain analyzed in this study. AL, axial length; LT, lens thickness; ACD, anterior chamber depth; PL, posterior length. Each line and the lightly shaded area of the same color represent mean ± standard deviation, respectively.

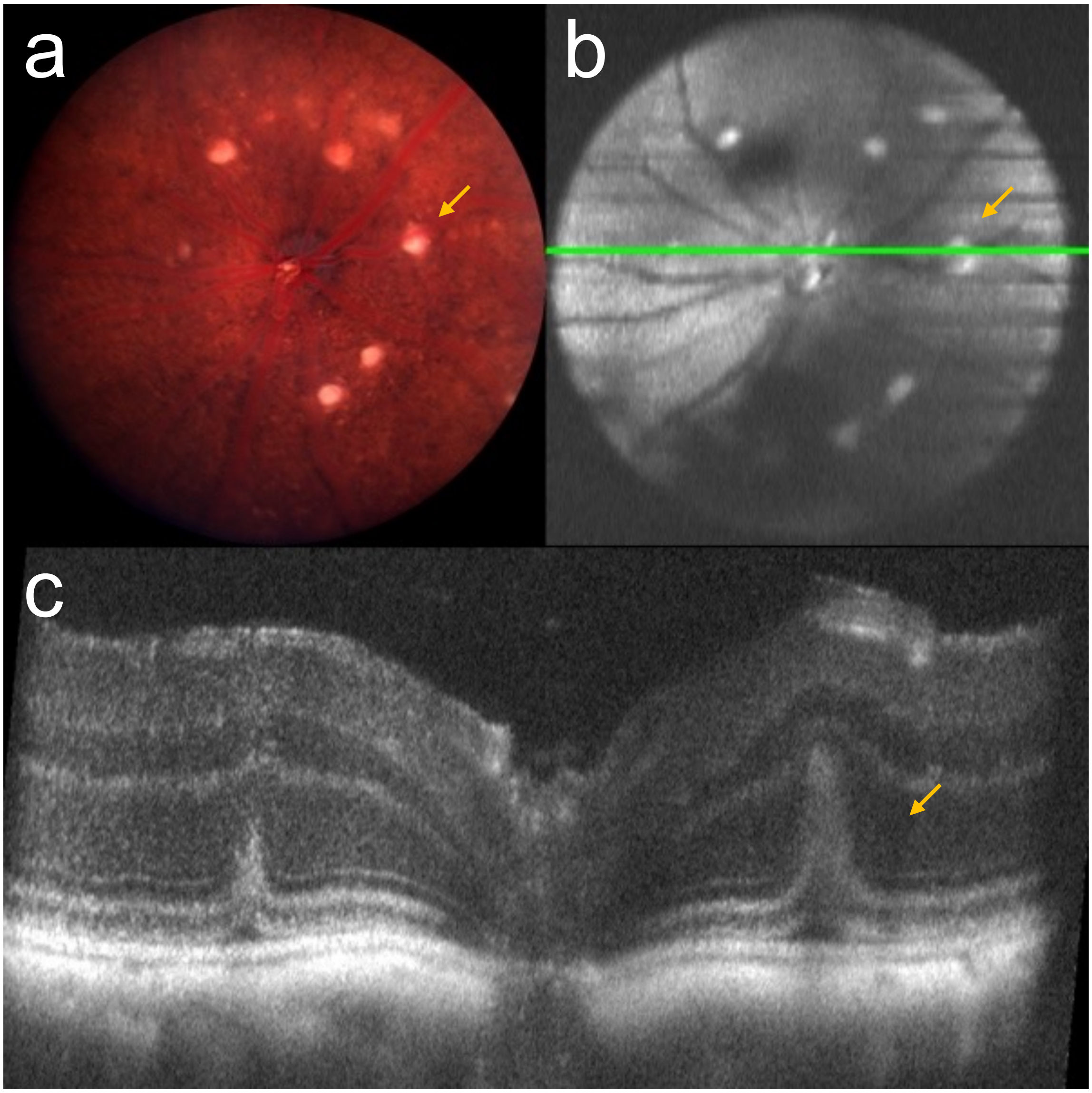

Figure S5: Abnormal ocular findings in 12-month-old *Prss56*^{glcr4} eyes. **a.** Fundus image (top left), **b.** OCT *en face* view (top right) and **c.** the corresponding B-scan (bottom panel) of the marked region (green line) in the *en face* image (also shown in Figure 8). Comparative analysis shows that the fundus spots (yellow arrow) correspond to the retinal folds observed by OCT.

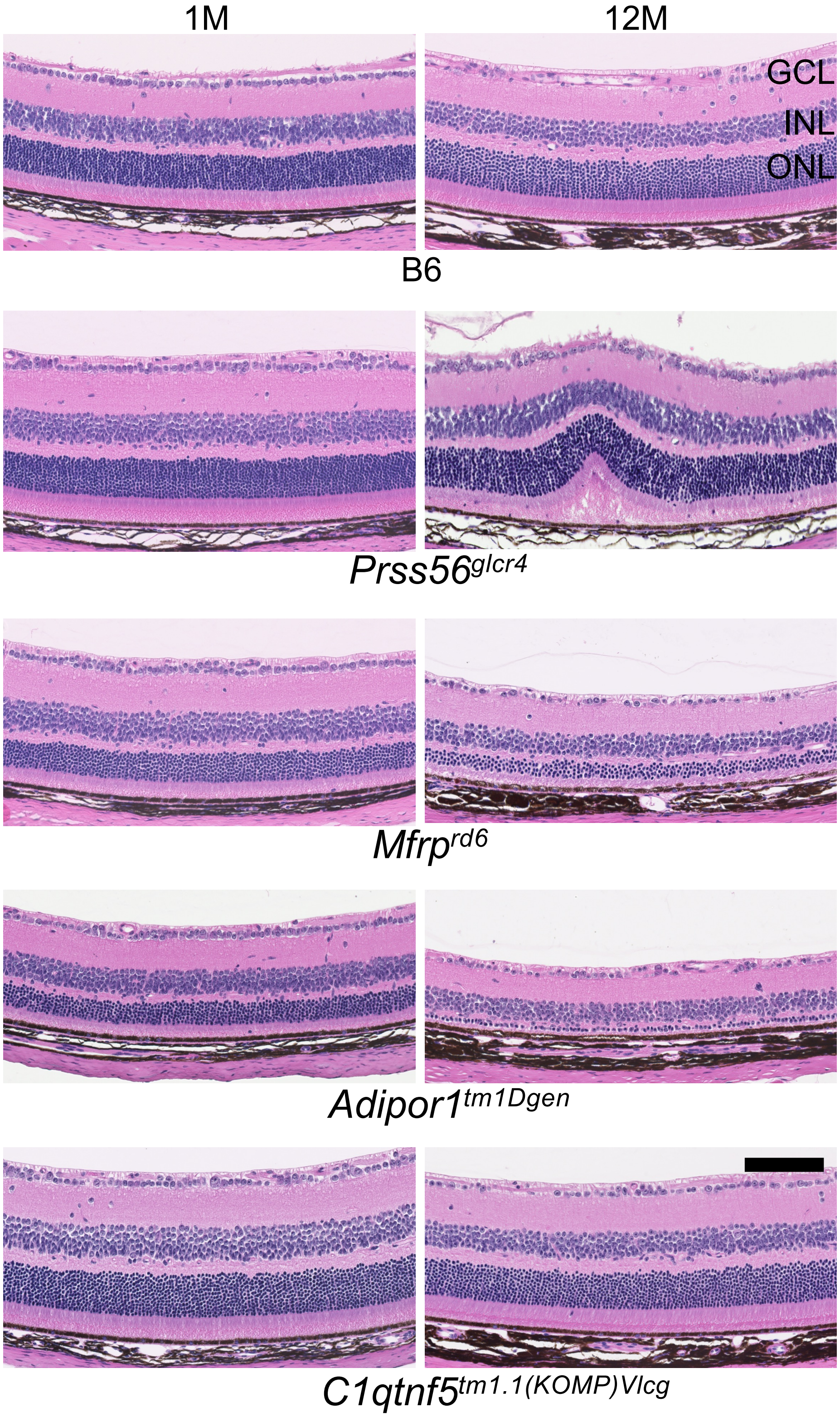

Figure S6 Retinal sections from B6 control and mutant mice, at 1 and 12 months of age, stained with hematoxylin and eosin (H&E), show a progressive and mutant-specific decrease in ONL thickness. n = 3 for each strain at each age. Scale – 100 μm.

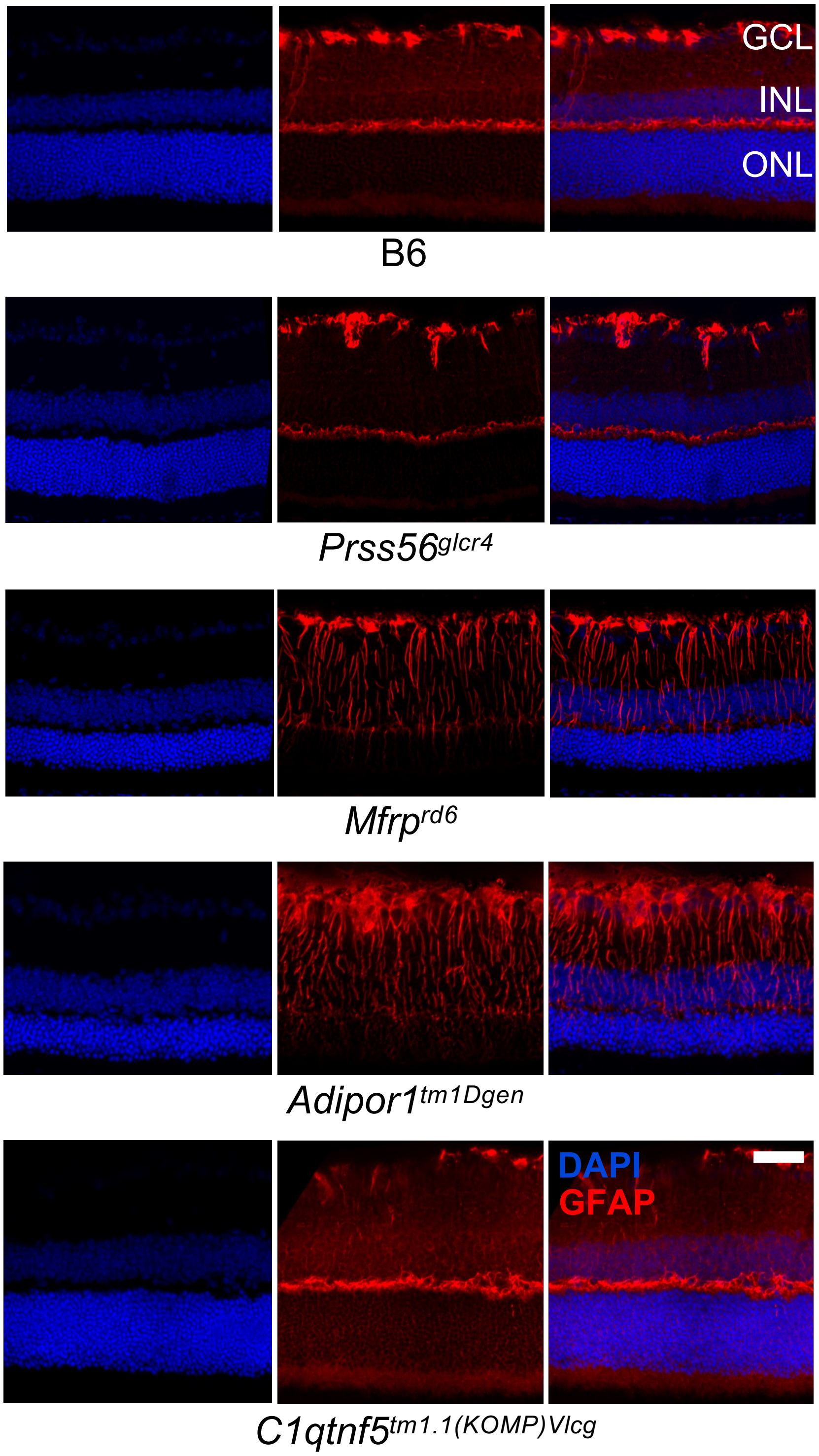

Figure S7: 4-month-old B6 and mutant mice retinal sections, stained with GFAP and DAPI. GFAP immunoreactivity was restricted to retinal astrocytes in B6, *Prss56*^{glcr4} and *C1qtnf5*$^{tm1.1(KOMP)Vlcg}$ mice. In the case of *Mfrp*^{rd6} and *Adipor1*^{tm1Dgen} mice, GFAP immunoreactivity, likely due to Müller cell activation in response to retinal degeneration, was also observed. n = 3 per strain. Scale – 100 μm.

Mice used in the study	Referred to as:
B6.C3Ga-*Mfrp*^{rd6}/J (*JAX*® Mice, stock 003684)	*Mfrp*^{rd6}
B6(Cg)-*Prss56*^{glcr4}/Sj	*Prss56*^{glcr4}
C57BL/6NJ-*Prss56*$^{em2(IMPC)J}$/Mmjax (MMRRC, stock 043778-JAX)	*Prss56*$^{em2(IMPC)J}$
B6.129P2-*Adipor1*^{tm1Dgen}/Mmnc (MMRRC, stock 011599-UNC)*	*Adipor1*^{tm1Dgen}
B6N(Cg)-*C1qtnf5*$^{tm1.1(KOMP)Vlcg}$/JMmucd (KOMP2, stock 018562-JAX available at MMRRC, stock 046844-UCD)	*C1qtnf5*$^{tm1.1(KOMP)Vlcg}$
C57BL/6J (*JAX*® Mice, stock 000664)	B6

Table ST1: Mice strains, and abbreviations used in this study. All mice strains were either already on B6 background or backcrossed to B6 for at least five generations and the *rd8* mutation was removed.

*The mouse strain used for this research project, B6.129P2*Adipor1*^{tm1Dgen}/Mmnc, RRID:MMRRC_011599-UNC, was obtained from the Mutant Mouse Resource and Research Center, an NIH-funded strain repository, and was donated to the MMRRC by Deltagen.

GENOTYPING					
**Strain**	**Allele**	**Size (bp)**	**Forward primers 5'>3'**	**Reverse primers 3'>5'**	**Assay***
Adipor1^{tm1Dgen}	WT	265	TTAGAGGCAGGGTAAGCTGAT	AGCCAGCTCCACTGTGTCAG	MCS
	MUT	~450		TGGGATTAGATAAATGCCTGCTCT	
C1qtnf5$^{tm1.1(KOMP)Vlcg}$	WT	382	CTACCTTTCGACCGTGTGCT	CCGGGTTCACTGTGTTTTAAG	S
	MUT	729	CGGTCGCTACCATTACCAGT	GGAGCAGCAGAGATGGAGTC	
Mfrp^{rd6}	WT	116	ACTACCACCCCAGCAAGGAC Probe:5HEX-CAGACCAGTAAGTCCCAAGGG	CTTTCCTCCCCAACACCATC Probe:6FAM-CAGACCAGTCCCAAGGGC	EP
	MUT	112			
Prss56^{glcr4}	WT	175	TGGCTCCAGAAACCAAAGCCGGA AGAGCGCCCGGAAACAAAGAGT	TCCTGGAAGAGAGGGAGTGA (Common)	AS
	MUT	152	GCGGCGCCCGGAAACAAAAGGA		
Prss56$^{em2(IMPC)J}$	WT	104	CCCAGGGTAGGAGAACATCA	GCCTGTAAGTGTGGCTGTTG (Common)	S
	MUT	120	GGTTGTACCAGAAGTGCTACCC		
qRT-PCR					
**Gene (primers)**	**Size (bp)**		**Forward**	**Reverse**	
C1qtnf5 (F1/R1)	126		GAGACCGGGACTACCTG	GATCGCTTGGCACTGAA	
C1qtnf5 (F2/R2)	117		GCACACCAGGTCACCAT	CAGGTAGTCCCGGTCTC	
Prss56 (F/1R)	183		CTGTGGACTGTGATGCTCGC	ACCCTGGGGAAGGCAAAT	
Actb (F/R)	207		CCAGTTCGCCATGGATGACGATAT	GTCAGGATACCTCTCTTGCTCTG	

Table ST2: Primers used for genotyping and qRT-PCR assay.

*AS, Allele-specific; EP, End-Point; MCS, Melting Curve; S, Standard PCR

Dunnett's multiple comparison	Adjusted *p*-value	Summary
**Age (1 month)**		
B6 vs. *Mfrp*^{rd6}	<0.0001	****
B6 vs. *Prss56*^{glcr4}	0.0015	**
B6 vs. *Adipor1*^{tm1Dgen}	0.1452	ns
B6 vs. *C1qtnf5*$^{tm1.1(KOMP)Vlcg}$	0.9965	ns
**Age (4 month)**		
B6 vs. *Mfrp*^{rd6}	<0.0001	****
B6 vs. *Prss56*^{glcr4}	<0.0001	****
B6 vs. *Adipor1*^{tm1Dgen}	0.0049	**
B6 vs. *C1qtnf5*$^{tm1.1(KOMP)Vlcg}$	0.4216	ns
**Age (8 month)**		
B6 vs. *Mfrp*^{rd6}	<0.0001	****
B6 vs. *Prss56*^{glcr4}	<0.0001	****
B6 vs. *Adipor1*^{tm1Dgen}	0.0002	***
B6 vs. *C1qtnf5*$^{tm1.1(KOMP)Vlcg}$	>0.9999	ns
**Age (12 month)**		
B6 vs. *Mfrp*^{rd6}	<0.0001	****
B6 vs. *Prss56*^{glcr4}	<0.0001	****
B6 vs. *Adipor1*^{tm1Dgen}	0.0006	***
B6 vs. *C1qtnf5*$^{tm1.1(KOMP)Vlcg}$	0.0228	*

Table ST3: Adjusted *p*-values obtained from Dunnett's multiple comparison test for AL comparison between different mouse strains, at four different ages. **** $p < 0.0001$, *** $p < 0.001$, ** $p < 0.01$ and * $p < 0.05$. ns, nonsignificant.

Dunnett's multiple comparison	Adjusted *p*-value	Summary
**Age (1 month)**		
B6 vs. *Mfrp*^{rd6}	0.5825	ns
B6 vs. *Prss56*^{glcr4}	0.2848	ns
B6 vs. *Adipor1*^{tm1Dgen}	0.0740	ns
B6 vs. *C1qtnf5*$^{tm1.1(KOMP)Vlcg}$	0.1680	ns
**Age (4 month)**		
B6 vs. *Mfrp*^{rd6}	0.0517	ns
B6 vs. *Prss56*^{glcr4}	0.8791	ns
B6 vs. *Adipor1*^{tm1Dgen}	0.9859	ns
B6 vs. *C1qtnf5*$^{tm1.1(KOMP)Vlcg}$	0.9906	ns
**Age (8 month)**		
B6 vs. *Mfrp*^{rd6}	0.0099	**
B6 vs. *Prss56*^{glcr4}	>0.9999	ns
B6 vs. *Adipor1*^{tm1Dgen}	0.8795	ns
B6 vs. *C1qtnf5*$^{tm1.1(KOMP)Vlcg}$	0.6797	ns
**Age (12 month)**		
B6 vs. *Mfrp*^{rd6}	0.9214	ns
B6 vs. *Prss56*^{glcr4}	0.8213	ns
B6 vs. *Adipor1*^{tm1Dgen}	0.4689	ns
B6 vs. *C1qtnf5*$^{tm1.1(KOMP)Vlcg}$	0.3177	ns

Table ST4: Adjusted *p*-values obtained from Dunnett's multiple comparison test for CCT comparison between different mouse strains, at four different ages. Symbols are defined as in Table ST3.

Dunnett's multiple comparison	Adjusted *p*-value	Summary
**Age (1 month)**		
B6 vs. $Mfrp^{rd6}$	0.0289	*
B6 vs. $Prss56^{glcr4}$	<0.0001	****
B6 vs. $Adipor1^{tm1Dgen}$	0.0001	***
B6 vs. $C1qtnf5^{tm1.1(KOMP)Vlcg}$	<0.0001	****
**Age (4 month)**		
B6 vs. $Mfrp^{rd6}$	<0.0001	****
B6 vs. $Prss56^{glcr4}$	<0.0001	****
B6 vs. $Adipor1^{tm1Dgen}$	0.0075	**
B6 vs. $C1qtnf5^{tm1.1(KOMP)Vlcg}$	0.0987	ns
**Age (8 month)**		
B6 vs. $Mfrp^{rd6}$	<0.0001	****
B6 vs. $Prss56^{glcr4}$	<0.0001	****
B6 vs. $Adipor1^{tm1Dgen}$	0.0399	*
B6 vs. $C1qtnf5^{tm1.1(KOMP)Vlcg}$	0.8002	ns
**Age (12 month)**		
B6 vs. $Mfrp^{rd6}$	<0.0001	****
B6 vs. $Prss56^{glcr4}$	<0.0001	****
B6 vs. $Adipor1^{tm1Dgen}$	<0.0001	****
B6 vs. $C1qtnf5^{tm1.1(KOMP)Vlcg}$	0.7932	ns

Table ST5: Adjusted *p*-values obtained from Dunnett's multiple comparison test for ACD comparison between different mouse strains, at four different ages. Symbols are defined as in Table ST3.

Dunnett's multiple comparison	Adjusted *p*-value	Summary
**Age (1 month)**		
B6 vs. $Mfrp^{rd6}$	0.8557	ns
B6 vs. $Prss56^{glcr4}$	0.2301	ns
B6 vs. $Adipor1^{tm1Dgen}$	0.0200	*
B6 vs. $C1qtnf5^{tm1.1(KOMP)Vlcg}$	0.0011	**
**Age (4 month)**		
B6 vs. $Mfrp^{rd6}$	0.6808	ns
B6 vs. $Prss56^{glcr4}$	0.3875	ns
B6 vs. $Adipor1^{tm1Dgen}$	0.5083	ns
B6 vs. $C1qtnf5^{tm1.1(KOMP)Vlcg}$	0.8805	ns
**Age (8 month)**		
B6 vs. $Mfrp^{rd6}$	0.9955	ns
B6 vs. $Prss56^{glcr4}$	0.0065	**
B6 vs. $Adipor1^{tm1Dgen}$	0.1020	ns
B6 vs. $C1qtnf5^{tm1.1(KOMP)Vlcg}$	0.2413	ns
**Age (12 month)**		
B6 vs. $Mfrp^{rd6}$	0.0998	ns
B6 vs. $Prss56^{glcr4}$	0.0017	**
B6 vs. $Adipor1^{tm1Dgen}$	0.0242	*
B6 vs. $C1qtnf5^{tm1.1(KOMP)Vlcg}$	0.1394	ns

Table ST6: Adjusted *p*-values obtained from Dunnett's multiple comparison test for LT comparison between different mouse strains, at four different ages. Symbols are defined as in Table ST3.

Dunnett's multiple comparison	Adjusted *p*-value	Summary
**Age (1 month)**		
B6 vs. *Mfrp*^{rd6}	<0.0001	****
B6 vs. *Prss56*^{glcr4}	0.1296	ns
B6 vs. *Adipor1*^{tm1Dgen}	<0.0001	****
B6 vs. *C1qtnf5*$^{tm1.1(KOMP)Vlcg}$	0.0413	*
**Age (4 month)**		
B6 vs. *Mfrp*^{rd6}	<0.0001	****
B6 vs. *Prss56*^{glcr4}	0.3345	ns
B6 vs. *Adipor1*^{tm1Dgen}	<0.0001	****
B6 vs. *C1qtnf5*$^{tm1.1(KOMP)Vlcg}$	0.7459	ns
**Age (8 month)**		
B6 vs. *Mfrp*^{rd6}	<0.0001	****
B6 vs. *Prss56*^{glcr4}	0.0018	**
B6 vs. *Adipor1*^{tm1Dgen}	<0.0001	****
B6 vs. *C1qtnf5*$^{tm1.1(KOMP)Vlcg}$	0.0438	*
**Age (12 month)**		
B6 vs. *Mfrp*^{rd6}	<0.0001	****
B6 vs. *Prss56*^{glcr4}	0.0407	*
B6 vs. *Adipor1*^{tm1Dgen}	<0.0001	****
B6 vs. *C1qtnf5*$^{tm1.1(KOMP)Vlcg}$	0.9760	ns

Table ST7: Adjusted *p*-values obtained from Dunnett's multiple comparison test for ONLT comparison between different mouse strains, at four different ages. Symbols are defined as in Table ST3.

Dunnett's multiple comparison	Adjusted *p*-value	Summary
**Age (1 month)**		
B6 vs. *Mfrp*^{rd6}	<0.0001	****
B6 vs. *Prss56*^{glcr4}	0.3047	ns
B6 vs. *Adipor1*^{tm1Dgen}	<0.0001	****
B6 vs. *C1qtnf5*$^{tm1.1(KOMP)Vlcg}$	<0.0001	****
**Age (4 month)**		
B6 vs. *Mfrp*^{rd6}	<0.0001	****
B6 vs. *Prss56*^{glcr4}	<0.0001	****
B6 vs. *Adipor1*^{tm1Dgen}	<0.0001	****
B6 vs. *C1qtnf5*$^{tm1.1(KOMP)Vlcg}$	0.1499	ns
**Age (8 month)**		
B6 vs. *Mfrp*^{rd6}	<0.0001	****
B6 vs. *Prss56*^{glcr4}	<0.0001	****
B6 vs. *Adipor1*^{tm1Dgen}	<0.0001	****
B6 vs. *C1qtnf5*$^{tm1.1(KOMP)Vlcg}$	0.2259	ns
**Age (12 month)**		
B6 vs. *Mfrp*^{rd6}	<0.0001	****
B6 vs. *Prss56*^{glcr4}	<0.0001	****
B6 vs. *Adipor1*^{tm1Dgen}	<0.0001	****
B6 vs. *C1qtnf5*$^{tm1.1(KOMP)Vlcg}$	0.6623	ns

Table ST8: Adjusted *p*-values obtained from Dunnett's multiple comparison test for RT comparison between different mouse strains, at four different ages. Symbols are defined as in Table ST3.

Dunnett's multiple comparison	Adjusted *p*-value	Summary
**Age (1 month)**		
B6 vs. $Mfrp^{rd6}$	<0.0001	****
B6 vs. $Prss56^{glcr4}$	<0.0001	****
B6 vs. $Adipor1^{tm1Dgen}$	0.2112	ns
B6 vs. $C1qtnf5^{tm1.1(KOMP)Vlcg}$	0.0745	ns
**Age (4 month)**		
B6 vs. $Mfrp^{rd6}$	<0.0001	****
B6 vs. $Prss56^{glcr4}$	<0.0001	****
B6 vs. $Adipor1^{tm1Dgen}$	0.0019	**
B6 vs. $C1qtnf5^{tm1.1(KOMP)Vlcg}$	0.9991	ns
**Age (8 month)**		
B6 vs. $Mfrp^{rd6}$	<0.0001	****
B6 vs. $Prss56^{glcr4}$	<0.0001	****
B6 vs. $Adipor1^{tm1Dgen}$	<0.0001	****
B6 vs. $C1qtnf5^{tm1.1(KOMP)Vlcg}$	0.0016	**
**Age (12 month)**		
B6vs. $Mfrp^{rd6}$	<0.0001	****
B6 vs. $Prss56^{glcr4}$	<0.0001	****
B6 vs. $Adipor1^{tm1Dgen}$	<0.0001	****
B6 vs. $C1qtnf5^{tm1.1(KOMP)Vlcg}$	<0.0001	****

Table ST9: Adjusted *p*-values obtained from Dunnett's multiple comparison test for VCD comparison between different mouse strains, at four different ages. Symbols are defined as in Table ST3.

Dunnett's multiple comparison	Adjusted *p*-value	Summary
**Age (1 month)**		
B6vs. $Mfrp^{rd6}$	<0.0001	****
B6 vs. $Prss56^{glcr4}$	<0.0001	****
B6 vs. $Adipor1^{tm1Dgen}$	0.0007	***
B6 vs. $C1qtnf5^{tm1.1(KOMP)Vlcg}$	0.8142	ns
**Age (4 month)**		
B6vs. $Mfrp^{rd6}$	<0.0001	****
B6 vs. $Prss56^{glcr4}$	<0.0001	****
B6 vs. $Adipor1^{tm1Dgen}$	<0.0001	****
B6 vs. $C1qtnf5^{tm1.1(KOMP)Vlcg}$	0.8898	ns
**Age (8 month)**		
B6 vs. $Mfrp^{rd6}$	<0.0001	****
B6 vs. $Prss56^{glcr4}$	<0.0001	****
B6 vs. $Adipor1^{tm1Dgen}$	<0.0001	****
B6 vs. $C1qtnf5^{tm1.1(KOMP)Vlcg}$	0.0002	***
**Age (12 month)**		
B6 vs. $Mfrp^{rd6}$	<0.0001	****
B6 vs. $Prss56^{glcr4}$	<0.0001	****
B6 vs. $Adipor1^{tm1Dgen}$	<0.0001	****
B6 vs. $C1qtnf5^{tm1.1(KOMP)Vlcg}$	<0.0001	****

Table ST10: Adjusted *p*-values obtained from Dunnett's multiple comparison test for PL comparison between different mouse strains, at four different ages. Symbols are defined as in Table ST3.

AL to	B6		*Mfrp^rd6*		*Prss56^glcr4*		*Adipor1^tm1Dgen*		*C1qtnf5^tm1.1(KOMP)Vlcg*	
	R	*P*	R	*P*	R	*P*	R	*P*	R	*P*
CT	0.2577	0.0300	0.1110	0.3601	0.3092	0.0087	0.3936	0.0004	0.5892	<0.0001
LT	0.9887	<0.0001	0.9872	<0.0001	0.9804	<0.0001	0.9835	<0.0001	0.9941	<0.0001
ACD	0.9599	<0.0001	0.9815	<0.0001	0.9725	<0.0001	0.9441	<0.0001	0.9725	<0.0001
ONLT	-0.4597	<0.0001	-0.9090	<0.0001	-0.5727	<0.0001	-0.9184	<0.0001	-0.7367	<0.0001
RT	-0.2510	<0.0001	-0.8857	<0.0001	0.1316	<0.0001	-0.8818	<0.0001	-0.8337	<0.0001
VCD	-0.9009	<0.0001	-0.9390	<0.0001	-0.9014	<0.0001	-0.8752	<0.0001	-0.9008	<0.0001
PL	-0.9054	<0.0001	-0.9467	<0.0001	-0.9111	<0.0001	-0.9334	<0.0001	-0.9102	<0.0001

Table ST11: Pearson's correlation coefficients (R) and *p* value obtained for identifying associations between AL and different axial and retinal parameters.

Dunnett's T3 multiple comparison test	Adjusted *p*-value	Summary
B6 vs. *Mfrp^rd6*	0.0051	**
B6 vs. *Prss56^glcr4*	<0.0001	****
B6 vs. *Adipor1^tm1Dgen*	0.0175	*
B6 vs. *C1qtnf5^tm1.1(KOMP)Vlcg*	<0.0001	****

Table ST12: Adjusted *p*-values obtained from Dunnett's multiple comparison test for CRC comparison between B6 and different mutant strains, at 4 months of age. Symbols are defined as in Table ST3.
